# Gender dependent modulation of opioid dependance genes and signaling pathways in HIV-1 Transgenic rats at morphine tolerance

**DOI:** 10.1007/s13365-025-01257-8

**Published:** 2025-05-07

**Authors:** Muhammed Bishir, Wenfei Huang, Ilker K. Sariyer, Sulie L. Chang

**Affiliations:** 1 Institute of NeuroImmune Pharmacology, Seton Hall University, South Orange, NJ 07079 USA; 2 Department of Microbiology, Immunology, and Inflammation, Center for Neurovirology and GeneEditing, Temple University Lewis Katz School of Medicine, Philadelphia, PA 19140 USA

**Keywords:** Morphine tolerance, QIAGEN knowledge base (QKB), Ingenuity pathway analysis (IPA), Neuroinflammation signaling pathway, CREB signaling in neurons, HIV-1Tg rats

## Abstract

Misuse of opioids is a major comorbidity in people with HIV (PWH). Neurological abnormalities and opioid addiction seen in PWH involve the interplay among signaling pathways. However, the impact of HIV proteins on morphine dependence is understudied. We aimed to understand the modulation of the opioid dependence genes and signaling pathways in the striatum (Str) and prefrontal cortex (PFC) of PWH. HIV-1 transgenic (HIV-1Tg) rats and F344 control animals were given 2 and 4 pellets of morphine (75-mg/pellet)/placebo on Days 1 and 2, respectively, via subcutaneous implantation. On Day 5, at morphine tolerance the rats were sacrificed, Str and PFC were collected for RNA isolation and cDNA preparation. A PCR-array was used to examine the expression of the 65 opioid dependance genes. Varying numbers of genes were significantly upregulated in the Str and PFC of morphine treated rats. Fold change values were uploaded to QIAGEN Ingenuity Pathway Analysis, to study the signaling pathways associated with the treatment conditions. CREB signaling in neurons and Neuroinflammation signaling pathway were highly activated in the Str of both male and female HIV-1Tg rats given morphine. Gαq signaling and S100 family signaling were activated in female HIV-1Tg rats received morphine. Similarly, in the PFC, synthesis of IP3, CREB Signaling in neurons, Gαq signaling in males and CREB Signaling in neuron, and Gαq signaling in females were activated. Using bioinformatic analysis, we identified key signaling pathways and gender dependent changes in the opioid dependent gene expression and pathway enrichment of HIV-1Tg rats at morphine tolerance.

**Supplementary Information:**

The online version contains supplementary material available at 10.1007/s13365-025-01257-8.

## Introduction

The human immunodeficiency virus (HIV) is a lentivirus that replicates by infecting and destroying primarily CD4 + T cells, a subset of lymphocytes that are essential for the normal function of the human immune system (Vigorito et al. 2015). In 2021, there are 38.4 million people living with HIV in the world, 1.5 million newly infected patients with 20% increase in the number of cases relative to 2010 (WHO, 2021). Since the advent of HIV, the HIV infection has been highly attributed to injection drugs including opioids (Hodder et al. 2021). Misuse of opioid substances, particularly morphine, has been identified as one of the major comorbidities in people with HIV (PWH) (Chang 2013). Morphine contributes to impairment of various functions like dysregulated macrophage functioning (Ko et al. 2019; Suzuki et al. 2002), increased neurocognitive functioning (Applebaum et al. 2009, 2010; Meyer et al. 2013) and alters the reward system in people living with HIV (PWH) (Chang et al. 2014; Reddy et al. 2012).

Morphine elicits its actions through the mu opioid receptors (MOR), which are widely distributed throughout the Central Nervous system and immune cells including lymphocytes and macrophages (Bidlack 2000; Chang et al. 2007; McCarthy et al. 2001). Our lab has previously shown that mRNA level of MOR was significantly higher in the peritoneal macrophages of HIV-1Tg rats than the control F344 rats. We have also shown that viral proteins including gp120 can upregulate MOR expression (Chang et al. 2007). Differential expressions of MOR and its signaling in HIV patients have a vital role in the increased neurological abnormalities. It is believed that enhanced neurocognitive dysfunction and increased addiction and dependance towards morphine seen in PWH is due to the interplay of overlapping signaling pathways.

In the last three decades, HIV-1 transgenic (HIV-1Tg) rats have been a well-accepted rodent model mimicking the HIV patients under combination Antiretroviral Therapy (cART) (Peng et al. 2010). The HIV-1Tg model was developed from an infectious clone of an integrated proviral plasmid (pNLS-3). An overlapping fragment containing 2 of the 9 viral genes, the gag gene at the 3′ region and the pol gene at the 5′ region, was deleted, resulting in a non-infectious provirus (pEVd1443). The gag gene is necessary for the assembly of HIV particles and the pol gene is required for morphological maturation of HIV particles. Thus, the HIV-1Tg rats lack viral assembly and replication (Vigorito et al. 2015). These rats show lower body weight (Peng et al. 2010), altered immune responses (Reid et al. 2001), altered immunophenotype (Abbondanzo and Chang 2014) impaired spatial learning and memory (Moran et al. 2013; Vigorito et al. 2007), impaired reward circuit (Bertrand et al. 2018) and neurocognitive deficits (McLaurin et al. 2017).

The mesolimbic dopamine reward circuit is central in regulating the development of addiction to various abusive drugs. The dopamine circuit is activated by majority of abusive drugs including morphine. Str is an integral part of the reward circuit; it receives input from various brain regions including PFC and Ventral Tegmental Area (VTA) to regulate decision making, motor planning, motivation and reward (Yager et al. 2015). In addition, the Str also receives glutaminergic inputs from cortex, amygdala, hippocampus and thalamus (Britt et al. 2012; Swanson 1982). The Str can be divided into two regions: the dorsal Str and the Nucleus Accumbens (NAc). Both the regions are highly innervated with dopaminergic neurons involved in addictive behavior (Everitt and Robbins 2005; Gerfen and Surmeier 2011). The NAc also receives projections from the PFC, the infralimbic region of the PFC projects towards NAc shell and the prelimbic region projects towards the NAc core (Britt et al. 2012; Sesack et al. 1989). Dysregulation of the PFC is commonly seen in drug abusers, and this results in the loss of inhibitory control and increased drug craving and relapse (Goldstein and Volkow 2011; Kalivas 2009). Also, drug induced neuroplasticity and behavior are modulated via the projections to the NAc shell (Ma et al. 2014). Both Str and PFC have a crucial role in addiction related events including drug seeking, craving, decision making and impaired inhibitory control. Reports have suggested HIV viral proteins impact on the neural process. In particular, Tat and gp120 target the fronto-striatal circuit and impair the dopamine signaling (Fitting et al. 2015). In addition, viral proteins have differential effects on the dopamine transporter (DAT) activity. Acute exposure to Tat blocks DAT activity and increase extracellular dopamine levels (Harrod et al. 2008; Zhu et al. 2009). Whereas chronic exposure to HIV viral proteins decreases the dopamine activity (Denton et al. 2019; Sinharay et al. 2017). Exposure to abusive drugs and HIV-1 leads to impaired dopamine signaling, and the fronto-striatal circuit. In both Str and PFC, the morphine’s effects on the mRNA transcript levels and the associated signaling pathways in the context of HIV-1 viral proteins is less studied.

Network-based meta-analysis have evolved greatly over the years, and they offer promising framework to identify signaling pathways, upstream regulators (molecules that are responsible for the observed gene expression change in the dataset) and diseases and functions. These in-silico tools have opened the wide possibilities of moving beyond “one gene”, one protein” perspective and exploring the multiple signaling pathways that are affected by the gene(s) expression changes. In the present study, we employed a novel approach by integrating in-vivo and in-silico approaches to identify the signaling pathways that are associated with the opioid dependance genes in the Str and PFC. QIAGEN Ingenuity Pathway Analysis (IPA) is a bioinformatics tool that employs a machine learning algorithm to identify the signaling pathways associated with the genes in the dataset from Qiagen Knowledge Base (QKB) (Bishir et al. 2023; Huang et al. 2020; Sarkar et al. 2013; Steven et al. 2020). QKB is a repository of over 7 million manually curated biological relationships that fuels QIAGEN IPA. QKB also has canonical pathways/signaling pathways, which are well-characterized metabolic signaling and cell signaling pathways. These pathways are manually hand drawn by scientists based on the millions of findings from literature and stored in the QKB. IPA’s algorithm identifies the presence of a gene from the uploaded dataset in a manually curated existing signaling pathway in the QKB. If majority of the genes in the uploaded dataset are present in a particular pathway, IPA’s algorithm predicts least p-value for the signaling pathway, suggesting the strong involvement of this signaling pathway in the observed gene expression changes. IPA’s core analysis identifies the canonical pathways/signaling pathways, upstream regulators and diseases and functions associated with the genes in the given dataset. It employs a right tailed Fisher’s exact test to calculate the probability of the occurrence of genes in the uploaded dataset in a curated signaling pathway in the QKB. Based on the number of genes, fold change and p-value of genes in the dataset, IPA computes a “p-value of overlap” for each signaling pathway. The p-value of overlap reflects the enrichment or association between the genes in the dataset and a curated signaling pathway in QKB.

Here we aimed to understand the modulation of the 65 opioid dependence genes and the signaling pathways in the Str and PFC of F344/HIV-1Tg rats during morphine tolerance. Both F344 and HIV-1Tg rats were sacrificed at their morphine tolerant stage, defined by reduction in the effects of morphine following its prolonged administration, tolerance leads to dependance, defined by a stage showing withdrawal effects when morphine’s dose is reduced or stopped and addiction characterized by drug seeking behavior (Eidson and Murphy 2019). The interplay between the opioid dependance genes at morphine’s tolerant state and associated overlapping signaling pathways could contribute to impaired reward circuit, opioid addiction and dependance attributed to people with HIV (PWH).

## Materials and methods

### Animals

Male and female Fischer F344 rats (3–4-week-old) were purchased from Envigo RMS, Inc. (Indianapolis, IN). Male and female HIV-1Tg rats were purchased from University of Maryland, Baltimore. The animals were housed in ventilated cages, 3 animals in each cage, in a temperature-controlled room (21–22 °C) with a 12-h light/12-h dark illumination cycle (lights-on at 7:00 AM). The animals were supplied the food and water ad libitum. The experimental protocol was approved by the Institutional Animal Care and Use Committee (IACUC) at Seton Hall University, South Orange, NJ.

### Morphine pelleting

Subcutaneous morphine pelleting was performed by employing a 2 + 4 morphine pelleting paradigm as described previously by (Rothman et al. 1986) and our publications (Mao et al. 2013; Zadina et al. 1995). In brief, following acclimatization, morphine pellets (75 mg of morphine sulfate) or placebo pellets were procured from the National Institute on Drug Abuse drug supply program (Rockville, MD). The placebo pellet was made of microcrystalline cellulose (NF 149.0 mg), colloidal silicon dioxide (NF 2.50 mg), and magnesium stearate (NF 1.50 mg). Both F344 and HIV-1Tg rats (male/female, 3–4 weeks old) were randomized to receive two pellets of either morphine or placebo on Day 1 and four pellets on Day 2 via subcutaneous (s.c.) implantation. The animals received 2 + 4 morphine pellets reaches tolerance on Day 5 as evident from tail flick latencies. Morphine treatment prior pelleting resulted in maximal latencies, whereas, on Day 5, morphine pelleted rats did not show morphine’s effect on tail flick latency (Rothman et al. 1986).

### RNA isolation and cDNA Preparation

Total RNA was extracted from Str and PFC using RNeasy Mini Kit (Qiagen, Germantown, MD), followed by RNase-free DNase (Qiagen) digestion to remove contaminating DNA. The RNA quality and quantity were determined using an ND1000 Nanodrop spectrophotometer (Thermo Scientific, Waltham, MA) and confirmed by gel electrophoresis. An equal amount of RNA (400 ng) from each sample was converted to cDNA using the RT2 First-Strand Kit (Qiagen, Germantown, MD) according to the manufacturer’s instructions.

### qRT-PCR analysis

Gene expression of opioid dependence genes was quantified using RT2 SYBR ROX qPCR Master Mix (Qiagen, Germantown, MD) as described previously (Chang et al. 2019; Liu et al. 2016). In brief, Reverse Transcription-quantitative Polymerase Chain Reaction (RT-qPCR) was performed with the QuantStudio 5 (Applied Biosystems, Foster, CA) using specific primers listed in Table 1. The thermocycler parameters were 95 °C for 10 min followed by 40 cycles at 95 °C for 15 s and 60 °C for 1 min. ROX was used as the passive reference. Expression of all 65 opioid dependance genes were normalized to expression of β-actin (Actb). The relative expression of each gene was compared to the respective controls using ΔΔCT method (Chang et al. 2019): F344 morphine vs. F344 placebo (morphine’s effect), HIV-1Tg rats morphine vs. HIV-1Tg rats-placebo (combined effect of morphine and HIV), and HIV-1Tg rat-placebo vs. F344 placebo (HIV effect). The primer sequences of the opioid dependance genes are listed in Table no: 1.

**Table 1:** PCR array primer sequence of 65 opioid dependance genes

Gene Symbol	Gene Name	Forward Primer (5’-->3’)	Reverse Prime *r*(5’-->3’)
Oprm1	opioid receptor mu 1	CAGCCCTTCCATGGTCACAG	TACTGGTCGCTAAGGGGTCTG
Oprm1	opioid receptor mu 1	ATCCAGTTCTTTACGCCTTCC	GATGTTCCCTAGTGTTCTGACG
Oprd1	opioid receptor delta 1	AGCTGGTACTGGGACACTGTGA	CACACGGTGATGATGAGAATGG
Oprk1	opioid receptor kappa 1	GGTGGGCTTAGTGGGCAAT	ATGTAGATGTTGGTTGCGGTCTT
Oprl1	opioid related nociceptin receptor 1	TGACCGCCATGAGCGTAGA	CTTTGCTGGATGTCCGAACA
Abat	4-aminobutyrate aminotransferase	GGAGGACCATGGGTTGCTTA	CAGTCAAAGGAAGGGATGTCAAT
Adh7	alcohol dehydrogenase 7 (class IV), mu or sigma polypeptide	CAGCCAAGATGCTCAGCTATGA	AAAGACGCAGCCCTTCCAT
Adk	adenosine kinase	AAGAAGCTCAAGGTGGAAGCG	CCACAGCAGAGATGTCAAGAAG
Adra1a	adrenoceptor alpha 1 A	GCATCGTGGTGGGTTGCT	TCCGGGAAGAAAGACCCAAT
Adra1b	adrenoceptor alpha 1B	TGTAGTCGGAATGTTCATCTTATGTTG	GGGCTTTAGGGTGGAGAACAG
Adra1d	adrenoceptor alpha 1D	GCCCTTCTCAGCCACTATGG	CCACACGTCGCAGAAGGTT
Adra2a	adrenoceptor alpha 2 A	GGCCTCAGCGGACATCCT	CATAACCTCGTTGGCCAAAGA
Adra2b	adrenoceptor alpha 2B	CAGCTCTTTGAACCCTGTCATCT	GGCCGGCAAAGGATCCT
Adra2c	adrenoceptor alpha 2 C	TGCGGCCTCAACGATGA	GGCAGGGCGCGAAGA
Aldh5a1	aldehyde dehydrogenase 5 family member A1	AACATGGCTTTTGTGTCTTTTCC	CTAGGGATAGTTCGGTGGTAGAACA
Avpr1a	arginine vasopressin receptor 1 A	TGAGAATTTCATCTGGACCGATT	CTGTTCAAGGAAGCCAGCAA
Cacna1b	calcium voltage-gated channel subunit alpha1 B	CCATCGTTCCCTACAGTTCCA	AGTACCGCATGGTCACAATGTAA
Cacna2d1	calcium voltage-gated channel auxiliary subunit alpha2delta 1	TTGCAGCTAGAGACATTGAGAAACTT	TGGGCGGCTTGGACTTT
Cacna2d2	calcium voltage-gated channel auxiliary subunit alpha2delta 2	GGAGATCCGTCGTAGTATGATTGA	TACCTCTCATCCAGGGATTTGAC
Chrna4	cholinergic receptor nicotinic alpha 4 subunit	GCCAGTAGCCAATATCTCAGATGTG	GTTCTTCTCGTCCACGTCAATG
Chrna10	cholinergic receptor nicotinic alpha 10 subunit	ACCTGGACGCAATCCGAAT	TCCGCCTTGTTGTAAAGTACGA
Cnr1	cannabinoid receptor 1	CAGGCCTTCCTACCACTTCATC	AAGTCAACAAAGCTGTACACAAAAATG
Cnr2	cannabinoid receptor 2	CAGAGAAGGTGGAGATACGGTTTC	CCCAAGATCACACAAGCAAGTG
Dbi	diazepam binding inhibitor, acyl-CoA binding protein	CAAGCTACTGTGGGCGATGTA	CACGAGTCCCACTTGGCTTT
Drd2	dopamine receptor D2	CAGGATTCACTGTGACATCTTTGTC	TGTGTTATACAGCATGGGCATTG
Drd3	dopamine receptor D3	TGCCATCAGCATAGACAGGTACA	TCATGAGTGCCACACGTCTACA
Drd4	dopamine receptor D4	ACACCCACCAACTACTTCATCGT	TGGACCTCGGAGTAGACAAAGAG
Gabra1	gamma-aminobutyric acid type A receptor alpha1 subunit	ACATGACCATGCCCAATAAACTC	TTCGGCTCTCACAGTCAACCT
Gabra3	gamma-aminobutyric acid type A receptor alpha3 subunit	TCACAAGTTTCTTTCTGGCTTAATAGAG	TCAAGGTGGTCATGGTGAGAAC
Gabra4	gamma-aminobutyric acid type A receptor alpha4 subunit	CTGTGCCTGGCGACTTGTT	TTTTCCGGGCACAATTTCTC
Gabra5	gamma-aminobutyric acid type A receptor alpha5 subunit	CATGCGCCTGACGATCTCT	TCCATCGGGAAGTCCTCAAG
Gabra6	gamma-aminobutyric acid type A receptor alpha6 subunit	TTTGGAATCACCACGGTTTTAA	TGCATAGGACACTTTGGGTAGAGA
Gabrb1	gamma-aminobutyric acid type A receptor beta1 subunit	GAAAAGAACAAACTGGAGATGAACAA	CACTGGTCTCATTCCTGATTTCC
Gabrb2	gamma-aminobutyric acid type A receptor beta2 subunit	CCCCGACTTGACTGATGTGA	AGACGATGTTGAAGAAGGAAAACAC
Gabrb3	gamma-aminobutyric acid type A receptor beta3 subunit	GGATCGAGCTCCCACAGTTC	TGTGGCGAAGACAACATTCC
Gabre	gamma-aminobutyric acid type A receptor epsilon subunit	TCCTGAGCAACTACGACCATAAACT	GTCCGAGGCTGTTGACAAAGA
Gabrg1	gamma-aminobutyric acid type A receptor gamma1 subunit	GACAGGAAGCTGAAAAGCAAAAC	GAAATGTTGTTCATGGGAATCAGA
Gabrg2	gamma-aminobutyric acid type A receptor gamma2 subunit	CAAGCAAGGATAAAGACAAAAAGAAGA	CATTTGGATCGTTGCTGATCTG
Gabrg3	gamma-aminobutyric acid type A receptor gamma3 subunit	CGACACCAGCAAGAACAACATTA	CCTGGCAATGGTGCTGAGT
Gabrp	gamma-aminobutyric acid type A receptor pi subunit	TCGGTACGTGGACTCGAAAAC	GATACGGTGACCAGGGTGAAA
Gad1	glutamate decarboxylase 1	GAGGAAATAGAGAGGTTGGGTCAA	AGGGCTATGGCCGGTGAT
Gad2	glutamate decarboxylase 2	GCCCCAAAGGAGATGTCAATTAT	AGTGGGCCTTTCTCCTTCACA
Grin1	glutamate ionotropic receptor NMDA type subunit 1	CACAGGAGCGGGTAAACAACA	TGAGTAGCTCGCCCATCATTC
Grin2a	glutamate ionotropic receptor NMDA type subunit 2 A	GCATCTGCCACAACGAGAAG	CCCGCCATGTTATCGATGTC
Grin2b	glutamate ionotropic receptor NMDA type subunit 2B	CTGTCCGCCTAGAGGTTTGG	TGCGCTGGGCTTCATCTT
Grin2c	glutamate ionotropic receptor NMDA type subunit 2 C	CTAGGAAGAACGGGCAGGAA	AGAGAACCTCCCCCTGCTATTC
Grin2d	glutamate ionotropic receptor NMDA type subunit 2D	GTCTGCACAGGTACTTCATGAACAT	GGTTCACCAGAAAGCCATCCT
Grin3a	glutamate ionotropic receptor NMDA type subunit 3 A	ATGACAACCAACGAAAATACATCTTT	TGAGGCAGGGAGCTCTCTTC
Grin3b	glutamate ionotropic receptor NMDA type subunit 3B	GGCATAGGGCTACCCCAAAA	AGCCTGAAGACTTGTACCTACTGATG
Hrh1	histamine receptor H1	CCCTGTTTCCGTCTCGACAT	CATCCATTTTGGGCTGGCTC
Htr1a	5-hydroxytryptamine receptor 1 A	CTCTGCTGGCTGCCGTTT	GCATGTGGCAGCTGTTTTCA
Htr2a	5-hydroxytryptamine receptor 2 A	TGATGTCACTTGCCATAGCTGATA	AGGCCACCGGTACCCATAC
Htr2b	5-hydroxytryptamine receptor 2B	GATCAACCCTGCCATGTACCA	GAGGAGAATGATGGATGAAGACTGA
Htr3a	5-hydroxytryptamine receptor 3 A	GGAAATGAAGTTCTACGTGGTCATC	GGAAGATACTGGGCAGCAAGA
Impdh1	inosine monophosphate dehydrogenase 1	GCATGGCTGTCAGGATATTGG	CCTCTATCTGGGCTGACATGGT
Impdh2	inosine monophosphate dehydrogenase 2	ACTGAGGCCCCTGGTGAGT	CAAGAGAGCCCATACCACGATAC
Scn10a	sodium voltage-gated channel alpha subunit 10	CATTGCTCCCTTCTGTTTGTGA	CCAACCCCTACGTGACTTCTCT
Slc6a2	solute carrier family 6 member 2	GCCTGGTGCTTCCAATGG	CATACCGTGGCCTCCTTGAG
Slc6a3	solute carrier family 6 member 3	CATGGATCCACTGCAACAACA	GGCCGTCGCTAGAGTTGCT
Slc6a4	solute carrier family 6 member 4	GCCATCAGCCCTCTGTTTCTC	AGTTGTATTGGAAAAGCCGTAGCT
Slc29a1	solute carrier family 29 member 1 (Augustine blood group)	CTCTTTAAGCATGATGTCTGGTTCA	GGCGAGGTAGCCATTGGA
Sigmar1	sigma non-opioid intracellular receptor 1	TATACCCTTCGCGCCTATGC	CCTGTCCACTCGCAGGTCTT
Hprt1	Hypoxanthine Phosphoribosyltransferase 1	GCCCTTGACTATAATGAGCACTTCA	GCCACATCAACAGGACTCTTGTAG
Actb	Actin Beta	TTCAACACCCCAGCCATGT	CAGAGGCATACAGGGACAACAC
Gapdh	Glyceraldehyde-3-Phosphate Dehydrogenase	AGAGAGAGGCCCTCAGTTGCT	TTGTGAGGGAGATGCTCAGTGT

### Ingenuity pathway analysis

The annual license for IPA software (https://www.qiagenbioinformatics.com/products) was purchased from QIAGEN Inc., Germantown, MD. The signaling pathways, upstream regulators and functions reported in the present study were obtained from the QIAGEN Knowledge Base (QKB). QKB is a repository of horizontally and vertically structured database composed of over 7 million individually modeled relationships between diseases, drugs, biological entities, such as genes, proteins, and metabolites, processes, such as expression, molecular cleavage, and phosphorylation, and the published results of omics experiments, such as increased or decreased expression. Sixty-five genes were identified to be involved in opioid dependence based on QKB. We employed IPA’s core analysis to identify the signaling pathways associated with the set of opioid dependance gene expression changes as described above in qRT-PCR Analysis. Fold change values and p-values of the opioid dependance genes in each treatment group was uploaded to IPA to identify the enriched canonical pathways/signaling pathway, upstream regulators and diseases and functions. IPA uses a network generation algorithm and right-tailed Fisher’s exact test to calculate the probability of the occurrence of genes in the uploaded dataset in a curated signaling pathway in the QKB. Based on the number of genes, gene expression fold changes and their p-values, IPA computes a p-value of overlap for each signaling pathway. A smaller p-value of overlap suggests greater enrichment of the signaling pathway. In addition, IPA calculates the activation z-score to infer the activation state (activation or inhibition) of the enriched pathways. A positive z-score indicates the activation of the pathway, whereas a negative z-score indicates inhibition. IPA’s upstream regulator analysis identifies upstream regulators and predicts their activation state in accordance with the gene expression changes in the uploaded dataset. Upstream regulators can be transcription factors, cytokines, enzymes, and endogenous molecules. We also employed pathway comparison analysis tool in IPA to compare the p-value of overlap of the signaling pathways and their activation z-scores among the treatment groups.

## Results

### Identification of opioid dependance genes

**Fig. 1:**
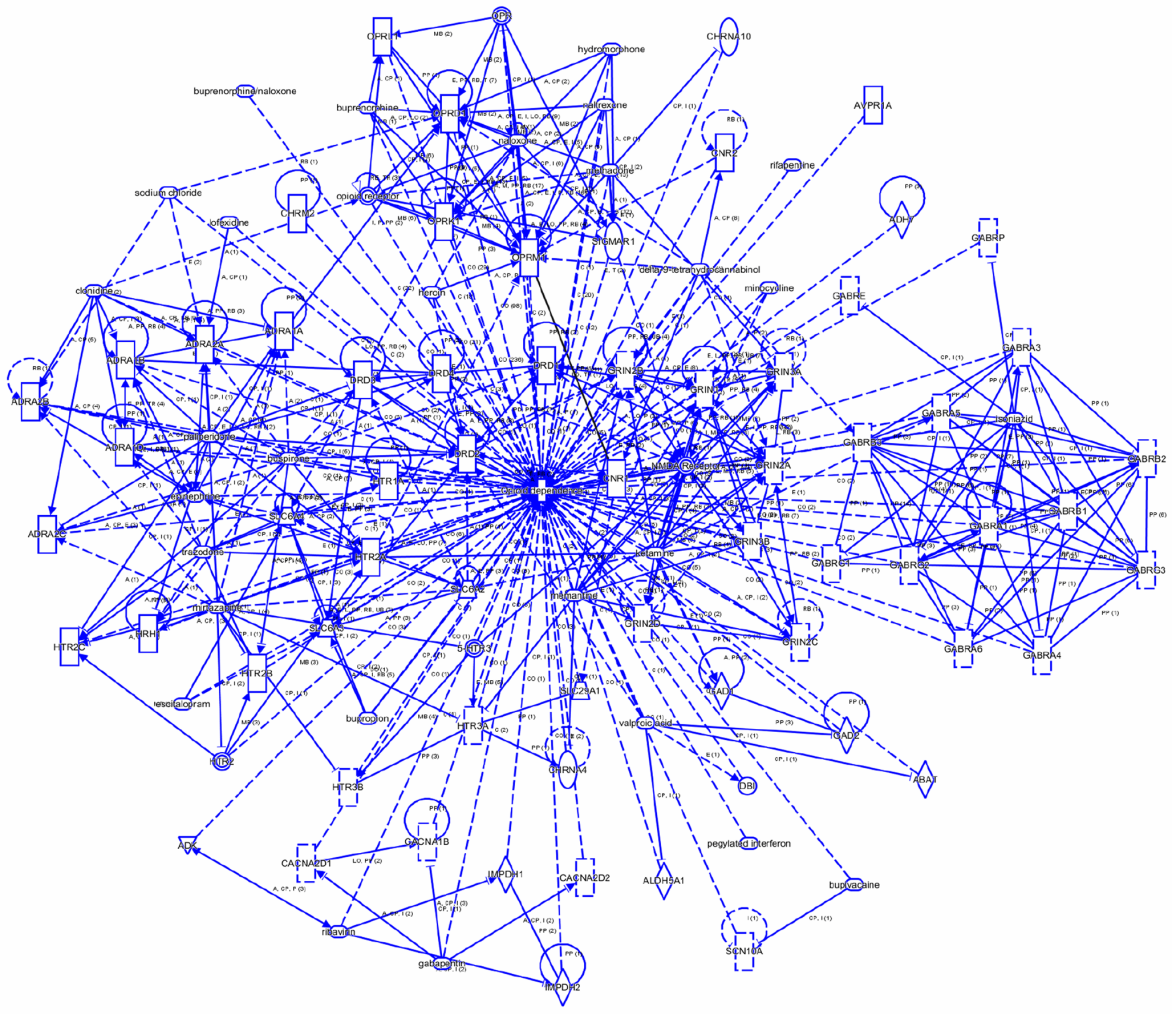
Interaction network of opioid dependance genes. IPA’s connect tool identified the interaction between 65 opioid dependance genes

Sixty-five genes involved in opioid dependence were identified from the QKB. These include, adrenoreceptors, arginine, vasopressin receptor, calcium voltage-gated channels, cholinergic receptors, cannabinoid receptors, dopamine receptors, glutamate decarboxylases, glutamate ionotropic receptor NMDA subunits, histamine receptor, 5-hydroxytryptamine receptors, inosine monophosphate dehydrogenases, opioid receptors, solute carrier family members. The list of the 65 genes with their symbols, Entrez Gene Name, Entrez Gene IDs for human, mouse, and rat was provided in Table 2. Network connectivity analysis of these 65 opioid dependance genes revealed that all these genes are highly interconnected (Fig. 1).

**Table 2:** List of opioid dependance genes

Symbol	Entrez Gene Name	Entrez Gene ID for Human	Entrez Gene ID for Mouse	Entrez Gene ID for Rat
ABAT	4-aminobutyrate aminotransferase	18	268,860	81,632
ADH7	alcohol dehydrogenase 7 (class IV), mu or sigma polypeptide	131	11,529	171,178
ADK	adenosine kinase	132	11,534	25,368
ADRA1A	adrenoceptor alpha 1 A	148	11,549	29,412
ADRA1B	adrenoceptor alpha 1B	147	11,548	24,173
ADRA1D	adrenoceptor alpha 1D	146	11,550	29,413
ADRA2A	adrenoceptor alpha 2 A	150	11,551	25,083
ADRA2B	adrenoceptor alpha 2B	151	11,552	24,174
ADRA2C	adrenoceptor alpha 2 C	152	11,553	24,175
ALDH5A1	aldehyde dehydrogenase 5 family member A1	7915	214,579	291,133
AVPR1A	arginine vasopressin receptor 1 A	552	54,140	25,107
CACNA1B	calcium voltage-gated channel subunit alpha1 B	774	12,287	257,648
CACNA2D1	calcium voltage-gated channel auxiliary subunit alpha2delta 1	781	12,293	25,399
CACNA2D2	calcium voltage-gated channel auxiliary subunit alpha2delta 2	9254	56,808	300,992
CHRM2	cholinergic receptor muscarinic 2	1129	243,764	
CHRNA4	cholinergic receptor nicotinic alpha 4 subunit	1137	11,438	25,590
CHRNA10	cholinergic receptor nicotinic alpha 10 subunit	57,053	504,186	64,574
CNR1	cannabinoid receptor 1	1268	12,801	25,248
CNR2	cannabinoid receptor 2	1269	12,802	57,302
DBI	diazepam binding inhibitor, acyl-CoA binding protein	1622	13,167	25,045
DRD1	dopamine receptor D1	1812	13,488	24,316
DRD2	dopamine receptor D2	1813	13,489	24,318
DRD3	dopamine receptor D3	1814	13,490	29,238
DRD4	dopamine receptor D4	1815	13,491	25,432
GABRA1	gamma-aminobutyric acid type A receptor alpha1 subunit	2554	14,394	29,705
GABRA3	gamma-aminobutyric acid type A receptor alpha3 subunit	2556	14,396	24,947
GABRA4	gamma-aminobutyric acid type A receptor alpha4 subunit	2557	14,397	140,675
GABRA5	gamma-aminobutyric acid type A receptor alpha5 subunit	2558	110,886	29,707
GABRA6	gamma-aminobutyric acid type A receptor alpha6 subunit	2559	14,399	29,708
GABRB1	gamma-aminobutyric acid type A receptor beta1 subunit	2560	14,400	25,450
GABRB2	gamma-aminobutyric acid type A receptor beta2 subunit	2561	14,401	25,451
GABRB3	gamma-aminobutyric acid type A receptor beta3 subunit	2562	14,402	24,922
GABRE	gamma-aminobutyric acid type A receptor epsilon subunit	2564		65,191
GABRG1	gamma-aminobutyric acid type A receptor gamma1 subunit	2565	14,405	140,674
GABRG2	gamma-aminobutyric acid type A receptor gamma2 subunit	2566	14,406	29,709
GABRG3	gamma-aminobutyric acid type A receptor gamma3 subunit	2567	14,407	79,211
GABRP	gamma-aminobutyric acid type A receptor pi subunit	2568	216,643	81,658
GAD1	glutamate decarboxylase 1	2571	14,415	24,379
GAD2	glutamate decarboxylase 2	2572	14,417	24,380
GRIN1	glutamate ionotropic receptor NMDA type subunit 1	2902	14,810	24,408
GRIN2A	glutamate ionotropic receptor NMDA type subunit 2 A	2903	14,811	24,409
GRIN2B	glutamate ionotropic receptor NMDA type subunit 2B	2904	14,812	24,410
GRIN2C	glutamate ionotropic receptor NMDA type subunit 2 C	2905	14,813	24,411
GRIN2D	glutamate ionotropic receptor NMDA type subunit 2D	2906	14,814	24,412
GRIN3A	glutamate ionotropic receptor NMDA type subunit 3 A	116,443	242,443	191,573
GRIN3B	glutamate ionotropic receptor NMDA type subunit 3B	116,444	170,483	170,796
HRH1	histamine receptor H1	3269	15,465	24,448
HTR1A	5-hydroxytryptamine receptor 1 A	3350	15,550	24,473
HTR2A	5-hydroxytryptamine receptor 2 A	3356	15,558	29,595
HTR2B	5-hydroxytryptamine receptor 2B	3357	15,559	29,581
HTR2C	5-hydroxytryptamine receptor 2 C	3358	15,560	25,187
HTR3A	5-hydroxytryptamine receptor 3 A	3359	15,561	79,246
HTR3B	5-hydroxytryptamine receptor 3B	9177	57,014	58,963
IMPDH1	inosine monophosphate dehydrogenase 1	3614	23,917	362,329
IMPDH2	inosine monophosphate dehydrogenase 2	3615	100,042,069|23,918	301,005
OPRD1	opioid receptor delta 1	4985	18,386	24,613
OPRK1	opioid receptor kappa 1	4986	18,387	29,335
OPRL1	opioid related nociceptin receptor 1	4987	18,389	29,256
OPRM1	opioid receptor mu 1	4988	18,390	25,601
SCN10A	sodium voltage-gated channel alpha subunit 10	6336	20,264	29,571
SIGMAR1	sigma non-opioid intracellular receptor 1	10,280	18,391	29,336
SLC29A1	solute carrier family 29 member 1 (Augustine blood group)	2030	63,959	63,997
SLC6A2	solute carrier family 6 member 2	6530	20,538	83,511
SLC6A3	solute carrier family 6 member 3	6531	13,162	24,898

### Protein-protein interaction network of opioid dependence genes and identification of clusters

**Fig. 2:**
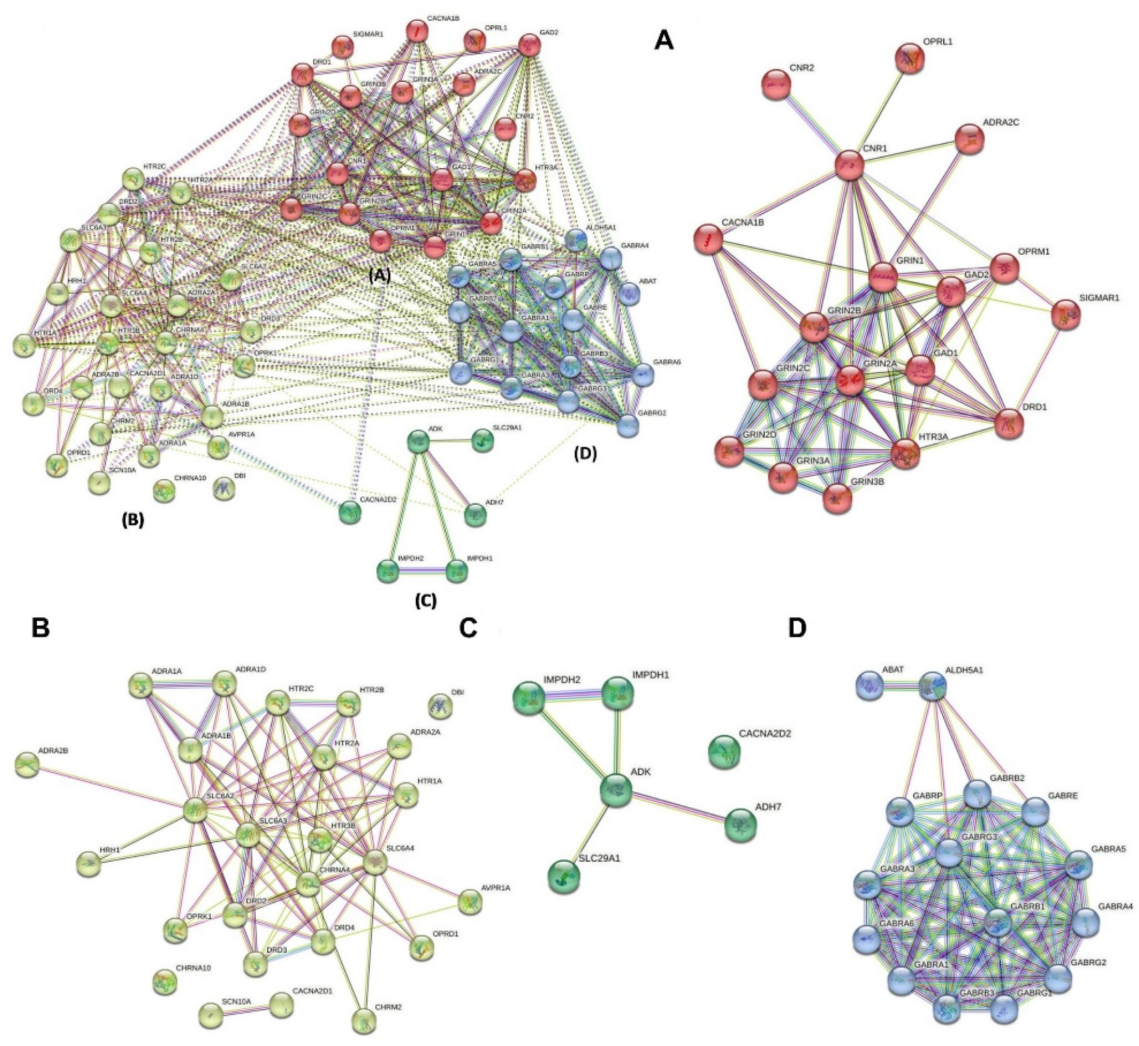
Protein-protein interaction network and gene clusters associated with 65 opioid dependance gene. Network interaction of 65 opioid dependance genes and the identified clusters (**A**, **B**, **C**, & **D**). The solid line indicates the interaction within the same cluster and the dotted line represents the intra cluster interactions

The Protein-protein interaction network of the 65 opioid dependence genes were constructed using the Search Tool for the Retrieval of Interacting Genes/Proteins (STRING) (Szklarczyk et al. 2019). As shown in Fig. 2, the network consists of 65 nodes and 437 edges. The analysis suggests a high clustering coefficient of 0.61 and average degree of ~ 13.4. The Adenylate cyclase inhibiting opioid receptor signaling pathway was found to be the top biological function associated with the network of 65 opioid dependance genes. The cluster analysis was performed using the K-means (Poswar Fde et al. 2015). The analysis resulted in four clusters (Fig. 2, A-D) with a maximum of 26 genes and a minimum of 6 genes. Cluster A had 18 genes and was found to be enriched in glutamate decarboxylation (Fig. 2A). There were 26 genes in Cluster B and they were found to be enriched in phospholipase C activating serotonin receptor signaling pathway, positive regulation of dopamine uptake involved in synaptic transmission, and adenylate cyclase inhibiting dopamine receptor signaling pathway (Fig. 2B). Cluster C had the least number of genes with only 6 genes, and they were mainly found to be enriched in GMP and GTP biosynthesis (Fig. 2C). Cluster D had 15 genes (Fig. 2D).

### Expression profile of opioid dependance genes in the Str and PFC of F344 and HIV-1Tg rats at morphine tolerance

A PCR array of 65 opioid dependance genes were performed to identify the expression level of these genes. Fold change values were calculated by comparing with respective controls, i.e., F344 Morphine vs. F344Placebo, HIV-1Tg Morphine vs. HIV-1Tg placebo, and HIV-1Tg placebo vs. F344 Placebo. In response to morphine, 12 genes including Adk, Adra2b, Adra2c, Cacan1b, Cacan2d1, Cnr2, Gabrb3, Grin2b, Grin2c, Grin2d, Grin3a, Impdh1 and Ifngr1 were found to be significantly (*p* < 0.05) upregulated in the Str of male F344 rats (Table 3A). In female F344 rats, expression 4 genes Gabra3, Gabra4, Gabrb1, Slc6a2 were significantly (*p* < 0.05) upregulated (Table 3B). In male HIV-1Tg rats, we observed significant upregulation of 15 genes (*p* < 0.05) including Abat, Adra2a, Adra2c, Avpr1a, Cacan2d2, Chrna10, Gabra4, Gabrg3, Gabrp, Gad2, Grin2c, Htr2b, Htr3a and Oprk1 (Table 4A). Whereas in female HIV-1Tg rats, only Cacan2d1 transcript level was significantly upregulated (*p* < 0.05) (Table 4B). There was no significant change in the expression of opioid dependence genes in the Str of placebo-treated HIV-1Tg rats.

In PFC of male F344 rats, transcript levels of 17 genes Abat, Adk, Adra1a, Aldh5a1, Cacan1b, Cacan2d1, Drd4, Gabra3, Gabra5, Gabrb2, Gabrp, Grin1, Grin2d, Grin3a, Htr2a, Impdh2, and Oprl1 were significantly upregulated at morphine tolerance (Table 5A). Whereas, in female F344 rats at morphine tolerance, only seven genes including Cacan2d1, Cnr2, Gabrb3, Grin2b, Grin2d, Oprl1, and Slc6a2 were significantly upregulated (*p* < 0.05) (Table 5B). In male HIV-1Tg rats, morphine treatment upregulated the expression of 11 genes: Adk, Aldh5a1, Cacan2d1, Gabrb1, Gabrb2, Gabrb3, Gabrg3, Grin2b, Grin2d, Htr3a and Slc6a2 (Table 6A). In female HIV-1Tg rats, 8 genes including Adra1a, Cnr1, Gabra5, Gabrb3, Grin2d, Grin3a, Htr2a and Slc6a2 were found to be significantly upregulated (*p* < 0.05) in response to morphine (Table 6B).

**Table 3:** Expression of significantly upregulated genes in the striatum of: (A) male F344 and (B) female F344 rats at morphine tolerance

**A) Male- F344 Morphine vs. F344Placebo**		
Gene	Fold Change	p-value
Adk	1.379	0.045
Adra2b	1.430	0.011
Adra2c	1.394	0.037
Cacan1b	1.417	0.005
Cacan2d1	1.849	0.026
Cnr2	1.616	0.016
Gabrb3	1.335	0.052
Grin2b	1.418	0.046
Grin2c	1.310	0.047
Grin2d	2.287	0.050
Grin3a	1.313	0.058
Impdh1	1.459	0.020
Ifngr1	1.478	0.037

**B) Female- F344 Morphine vs. F344Placebo**		
Gene	Fold Change	p-value
Gabra3	1.271	0.048
Gabra4	0.833	0.011
Gabrb1	1.337	0.011
Slc6a2	1.437	0.053

**Table 4:** Expression of significantly upregulated genes in the striatum of: (A) male HIV-1Tg and (B) female HIV-1Tg rats at morphine tolerance

**A) Male HIV-1Tg Morphine vs HIV-1Tg placebo**		
Gene	Fold Change	p-value
Abat	2.527	0.031
Adra2a	1.434	0.001
Adra2c	2.064	0.044
Avpr1a	2.350	0.015
Cacan2d2	3.915	0.006
Chrna10	3.619	0.05
Gabra4	1.429	0.007
Gabrg3	1.977	0.021
Gabrp	2.624	0.021
Gad2	2.551	0.027
Grin2c	1.475	0.008
Htr2b	1.514	0.045
Htr3a	1.497	0.020
Oprk1	3.953	0.038

**B) Female HIV-1Tg Morphine vs. HIV-1Tg placebo**		
Gene	Fold Change	p-value
Cacan2d1	1.265	0.047

**Table 5:** Expression of significantly upregulated genes in the PFC of: (A) male F344 and (B) female F344 rats at morphine tolerance

**A) Male- F344 Morphine vs. F344Placebo**		
Gene	Fold Change	p-value
Abat	1.768	0.042
Adk	1.821	0.003
Adra1a	1.431	0.000
Aldh5a1	1.712	0.003
Cacan1b	1.536	0.008
Cacan2d1	1.579	0.000
Drd4	0.618	0.015
Gabra3	1.583	0.001
Gabra5	1.442	0.042
Gabrb2	1.398	0.015
Gabrp	1.875	0.002
Grin1	1.388	0.004
Grin2d	1.675	0.005
Grin3a	1.773	0.001
Htr2a	1.251	0.047
Impdh2	1.580	0.010
Oprl1	1.297	0.001

**B) Female- F344 Morphine vs F344Placebo**		
Gene	Fold Change	p-value
Cacan2d1	1.231	0.046
Cnr2	1.032	0.005
Gabrb3	1.096	0.056
Grin2b	1.098	0.044
Grin2d	1.357	0.037
Oprl1	1.349	0.036
Slc6a2	1.371	0.033

**Table 6:** Expression of significantly upregulated genes in the PFC of: male HIV-1Tg and (B) female HIV-1Tg rats at morphine tolerance

**A) Male HIV-1Tg Morphine vs HIV-1Tg placebo**		
Gene	Fold Change	p-value
Adk	1.292	0.013
Aldh5a1	1.219	0.031
Cacan2d1	1.157	0.025
Gabrb1	1.280	0.029
Gabrb2	1.145	0.012
Gabrb3	1.232	0.023
Gabrg3	1.431	0.008
Grin2b	1.237	0.001
Grin2d	1.559	0.007
Htr3a	1.254	0.025
Slc6a2	1.419	0.050

**B) Female HIV-1Tg Morphine vs. HIV-1Tg placebo**		
Gene	Fold Change	p-value
Adra1a	1.333	0.014
Cnr1	1.294	0.058
Gabra5	1.608	0.009
Gabrb3	1.213	0.042
Grin2d	1.250	0.059
Grin3a	1.444	0.053
Htr2a	1.289	0.044
Slc6a2	1.381	0.051

### IPA’s core analysis identified the top signaling pathways, upstream regulators and functions in F344/HIV-1Tg rats at Morphine’s tolerance

**Fig. 3:**
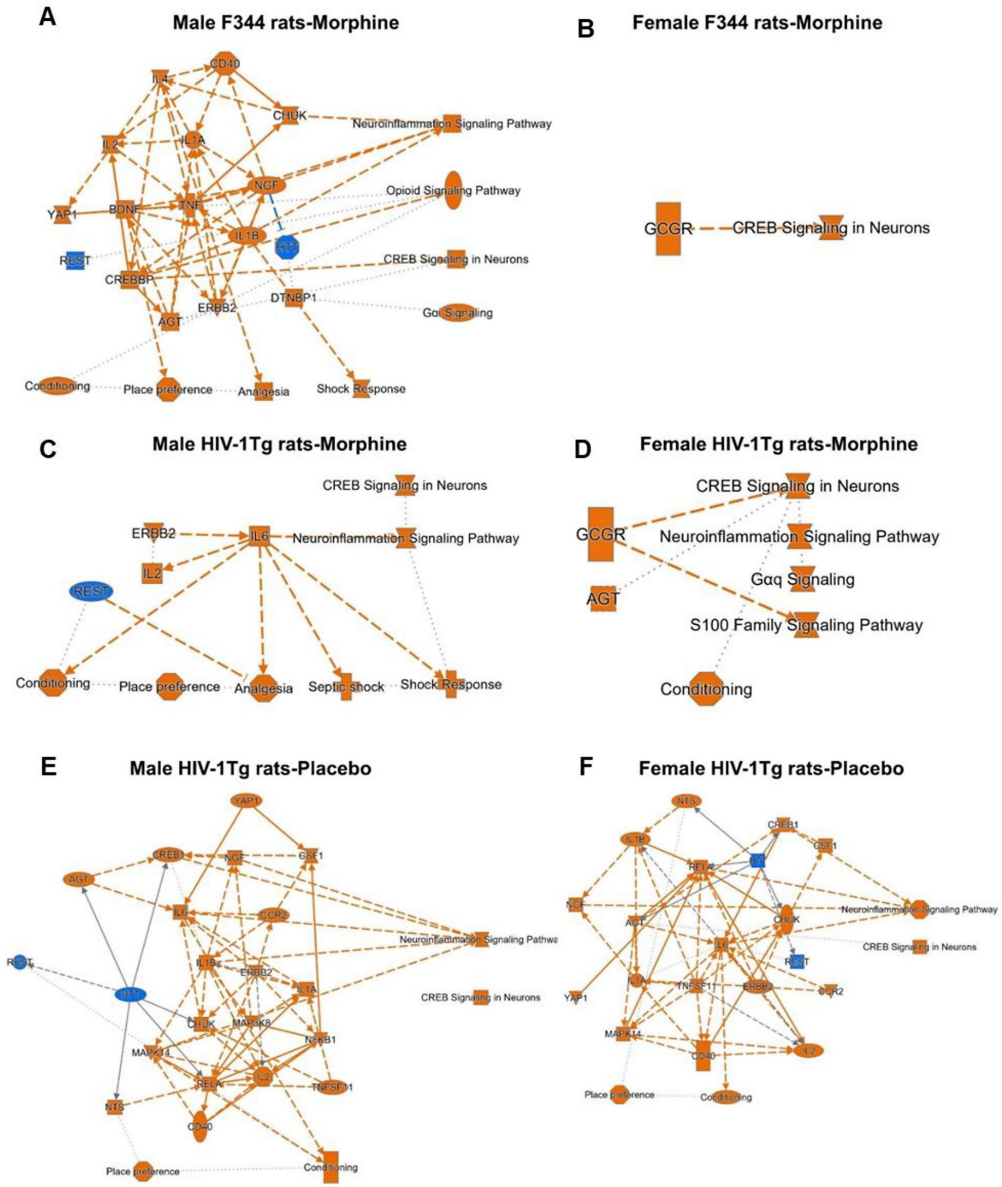
Graphical summary of the core analysis of the 65 opioid dependance genes in the striatum. (**A**) Male F344 morphine, (**B**) Female F344 morphine, (**C**) Male HIV-1Tg rat morphine, (**D**) Female HIV-1Tg rat morphine, (**E**) Male HIV-1Tg rat placebo, and (**F**) Female HIV-1Tg rat placebo

IPA’s core analysis examined the expression changes of morphine dependance genes in the Str of the F344 and HIV-1Tg rats, revealing the enriched signaling pathways and the upstream regulators. The graphical summary of the enriched pathways, upstream regulators and functions is given in Fig. 3. In male F344 rats received morphine pellets, Neuroinflammation signaling pathway, CREB signaling in neurons, Opioid signaling pathway, and Gαi signaling was found to be the top activated signaling pathway. Functions including analgesia, conditioning, and place preference were also activated. The activation of the signaling pathways and functions were regulated by the upstream regulators including CREBP, BDNF, inflammatory cytokines: IL4, IL2, and IL1A, NGF, ERBB2, angiotensinogen (AGT), YAP1, CHUK (activated); HTT and REST (inhibited) (Fig. 3A). In female F344 rats received morphine pellets, CREB signaling in neurons was found to the top signaling pathway activated, the activation was mediated by the upstream regulator glucagon receptor (GCGR) (Fig. 3B).

In male HIV-1Tg rats received morphine pellets, Neuroinflammation signaling pathway, and CREB signaling in neurons were the activated signaling pathways mediated via IL6 activation. Upstream regulators like IL2, and ERBB2 (activated) Re1 silencing transcription (REST) (inhibited) were the other top enriched upstream regulators. IL6 activation also mediated the activation of functions including conditioning, place preference, analgesia, and shock responses (Fig. 3C). In female HIV-1Tg rats received morphine pellets, Neuroinflammation signaling pathway, CREB signaling in neurons, Gαq signaling neurons, and S100 family signaling pathway were found to be activated. The activation was mediated via upstream regulators GCGR, and AGT (Fig. 3D).

In male HIV-1Tg rats received placebo, Neuroinflammation signaling pathway, and CREB signaling in neurons were the top activated signaling pathways mediated via upstream regulators including transcription regulators (CREB1, BDNF, NFkB1, RELA), inflammatory cytokines (IL4, IL2, and IL1A, TNFSF11), growth factors: NGF, AGT, Kinases (MAPK14, MAP3K8, ERBB2), GPCR (CCR2) and transmembrane receptor (CD40) (Fig. 3E). In female HIV-1Tg rats, Neuroinflammation signaling pathway, and CREB signaling in neurons were the top activated signaling pathways. Upstream regulators including transcription regulators (CREB1, YAP1, RELA), inflammatory cytokines (CSF1, IL2, IL6, IL1A, IL1B, TNFSF11), growth factors (NGF, AGT), Kinases: (MAPK14, ERBB2), GPCR (CCR2) and transmembrane receptor (CD40) were found to be activated and transcription regulators (HTT and REST) were inhibited (Fig. 3F).

**Fig. 4:**
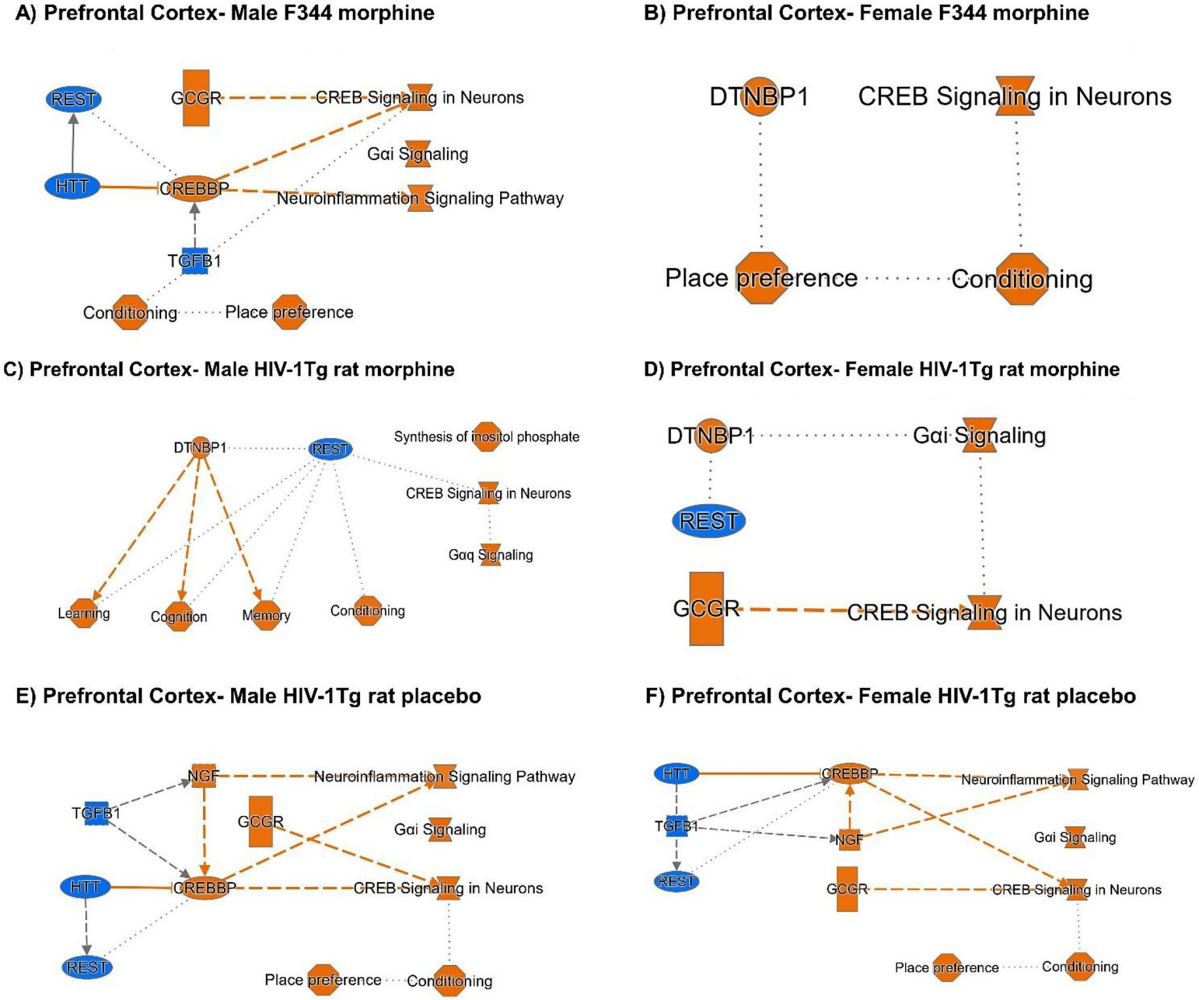
Graphical summary of the core analysis of the 65 opioid dependance genes in the PFC. (**A**) Male F344 morphine, (**B**) Female F344 morphine, (**C**) Male HIV-1Tg rat morphine, (**D**) Female HIV-1Tg rat morphine, (**E**) Male HIV-1Tg rat placebo, and (**F**) Female HIV-1Tg rat placebo

In the PFC of male F344 rats, CREB signaling in neurons, Gαi signaling and NeuroInflammation signaling pathway and functions like Conditioning and place preference were found to be activated. The activation of signaling pathways and functions were mediated by the activation of GCGR, and CREBP and inhibition of REST, HTT and TGFB1 upstream regulators (Fig. 4A). In females, CREB signaling in neurons was the top activated signaling pathway and functions like place preference and conditioning were found to be activated. The activation was mediated by the activation of Dysbindin (DTNBP1) (Fig. 4B).

In morphine-treated HIV-1Tg rats, synthesis of inositol phosphate, CREB signaling in neurons, and Gαq signaling, and functions like learning, cognition, memory and conditioning were found to be the top activated signaling pathways, mediated via the activation of DTNBP1 and inhibition of REST (Fig. 4C). In females, the activation of CREB signaling in neurons and Gαi signaling was mediated via the activation of GCGR and DTNBP1 respectively. Upstream regulator REST was found to be inhibited (Fig. 4D).

In the PFC of placebo-treated male and female HIV-1Tg rats, the activation of Neuroinflammation signaling pathway, Gαi signaling, and CREB signaling in neurons and functions including place preference and conditioning were mediated via the activation of NGF, CREBP and GCGR. Inhibition of TGFB1, HTT and REST regulates the activation of NGF and CREBP (Fig. 4E and F).

### Identification of significantly enriched overlapping signaling pathways associated with F344 and HIV-1Tg rats received morphine and HIV-1Tg rats received placebo pellets

**Fig. 5:**
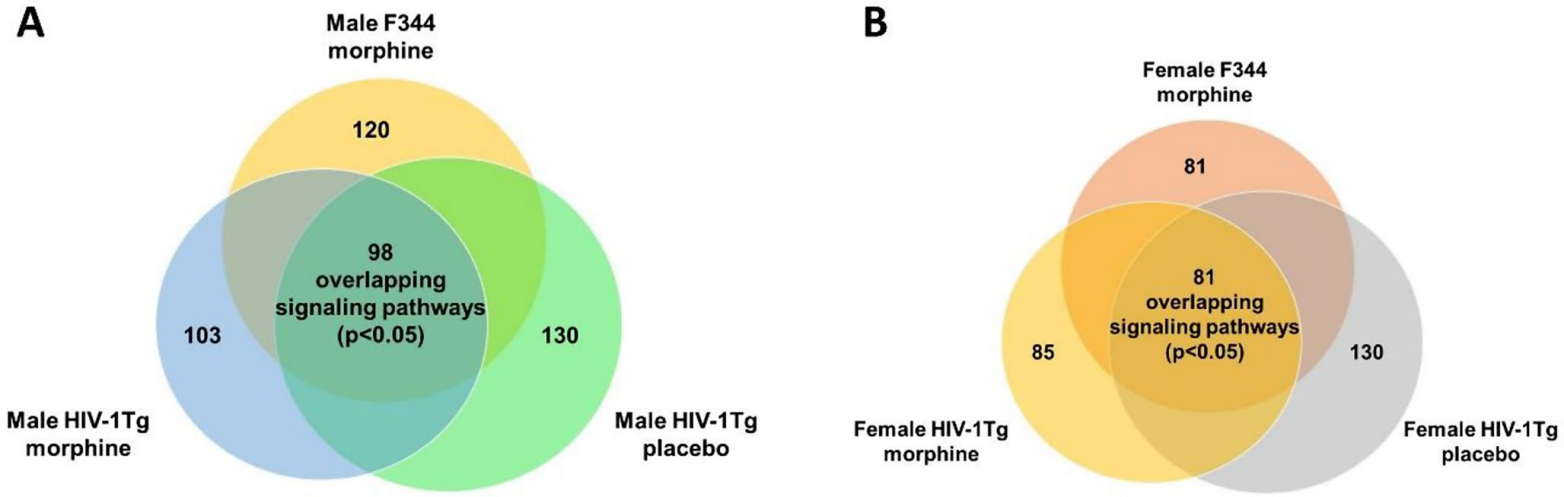
Significantly enriched signaling pathways in the striatum among the treatment groups. (**A**) Male: 98 commonly shared signaling pathways were found between the treatment groups. (**B**) Female: 81 commonly shared signaling pathways were found between the treatment groups

Core analysis of the 65 opioid dependance genes identified top enriched signaling pathways. In the Str of morphine-treated F344 rats, there were 120 significantly enriched signaling pathways (*p* < 0.05), and 103 significantly enriched signaling pathways in HIV-1Tg rats. In placebo- treated HIV-1Tg rats, 130 signaling pathways were found to be significantly enriched. There were 98 signaling pathways found to be enriched in all treatment groups (Fig. 5A). Among the 98 signaling pathways we found 27 signaling pathways that are closely involved in morphine dependance. These pathways include Neuroinflammation Signaling Pathway, Neurovascular Coupling Signaling Pathway, GABA Receptor Signaling, CREB Signaling in Neurons, S100 Family Signaling Pathway, G-Protein Coupled Receptor Signaling, Gαi Signaling, Phagosome Formation, Opioid Signaling Pathway, cAMP-mediated signaling, nNOS Signaling in Neurons, Reelin Signaling in Neurons, Dopamine-DARPP32 Feedback in cAMP Signaling, Glutamate Receptor Signaling, Neuropathic Pain Signaling In Dorsal Horn Neurons, Serotonin Receptor Signaling, Synaptic Long Term Potentiation, IL-10 Signaling, Gαq Signaling, Ephrin Receptor Signaling, AMPK Signaling, IL-6 Signaling, Pathogen Induced Cytokine Storm Signaling Pathway, IL-17 A Signaling, IL-12 Signaling and Production in Macrophages, IL-17 Signaling and Glucocorticoid Receptor Signaling. Similarly, in females, IPA’s core analysis identified 81 signaling pathways (*p* < 0.05) in F344 rats given morphine, 130 signaling pathways (*p* < 0.05) in HIV-1Tg-placebo rats and 85 signaling pathways (*p* < 0.05) in HIV-1Tg rats given morphine, among the 85 signaling pathways, 81 signaling pathways were overlapping with pathways in F344-morphine and HIV-1Tg-placebo group (Fig. 5B). The 27 signaling pathways mentioned above were also significantly enriched in female F344/HIV-1Tg rats (Table 7).

**Fig. 6:**
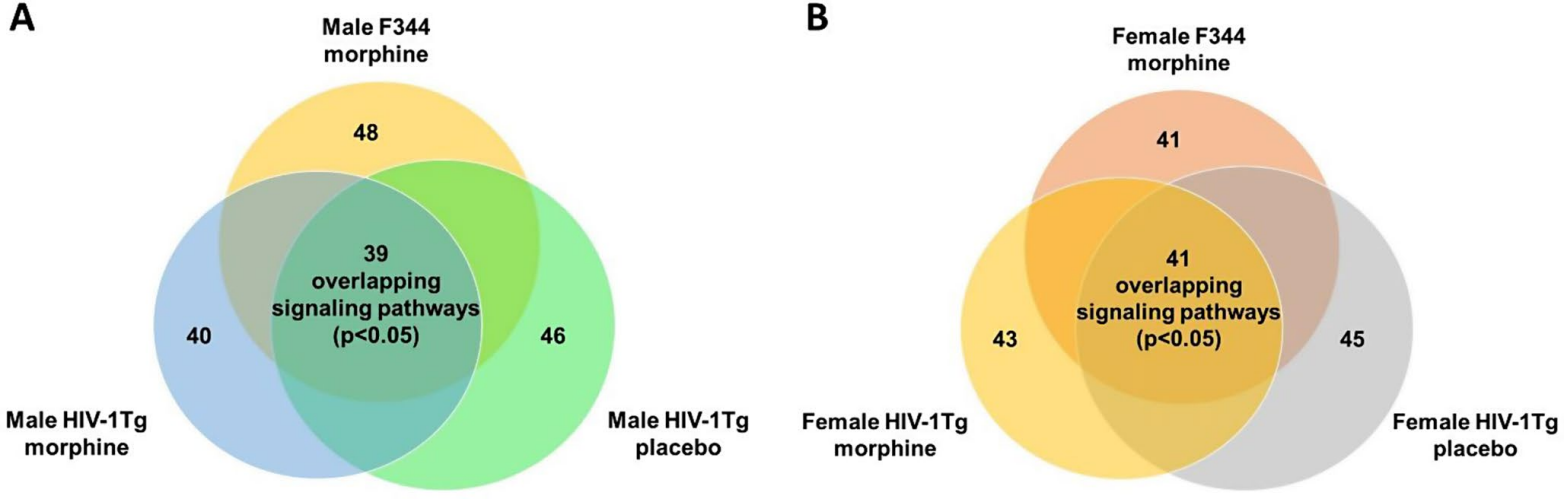
Significantly enriched signaling pathways in the PFC among the treatment groups. (**A**) Male: 39 commonly shared signaling pathways were found between the treatment groups. (**B**) Female: 41 commonly shared signaling pathways were found between the treatment groups

In the PFC, there were 48 significantly enriched signaling pathways (*p* < 0.05) in morphine treated F344 rats, and 40 significantly enriched signaling pathways in HIV-1 Tg rats. In placebo-treated male HIV-1Tg rats, 46 signaling pathways were found to be significantly enriched. 39 signaling pathways were found to be enriched in all treatment groups (Fig. 6A). In females, F344 morphine-treated group had 41 significantly enriched signaling pathways (*p* < 0.05), HIV-1Tg morphine group had 43 significantly enriched signaling pathways, and HIV-1Tg placebo treated group had 45 signaling pathways. There were 41 overlapping signaling pathways found to be overlapping in all treatment groups (Fig. 6B). The overlapping signaling pathways from both male and female treatment groups were further analyzed to identify the morphine addiction/dependance related pathways. We found that among the 27 signaling pathways found in the Str, 20 signaling pathways were present in the PFC (Table 8). These pathways include Neuroinflammation Signaling Pathway, Neurovascular Coupling Signaling Pathway, GABA Receptor Signaling, CREB Signaling in Neurons, S100 Family Signaling Pathway, G-Protein Coupled Receptor Signaling, Gαi Signaling, Phagosome Formation, Opioid Signaling Pathway, cAMP-mediated signaling, nNOS Signaling in Neurons, Reelin Signaling in Neurons, Dopamine-DARPP32 Feedback in cAMP Signaling, Glutamate Receptor Signaling, Neuropathic Pain Signaling In Dorsal Horn Neurons, Serotonin Receptor Signaling, Synaptic Long Term Potentiation, Gαq Signaling, Ephrin Receptor Signaling, and AMPK Signaling pathway.

**Table 7:** Signaling pathways associated with morphine and HIV in the Str

Sl No:	Signaling Pathways- Striatum
1	Neuroinflammation Signaling Pathway
2	Neurovascular Coupling Signaling Pathway
3	GABA Receptor Signaling
4	CREB Signaling in Neurons
5	S100 Family Signaling Pathway
6	G-Protein Coupled Receptor Signaling
7	Gαi Signaling
8	Phagosome Formation
9	Opioid Signaling Pathway
10	cAMP-mediated signaling
11	nNOS Signaling in Neurons
12	Reelin Signaling in Neurons
13	Dopamine-DARPP32 Feedback in cAMP Signaling
14	Glutamate Receptor Signaling
15	Neuropathic Pain Signaling In Dorsal Horn Neurons
16	Serotonin Receptor Signaling
17	Synaptic Long Term Potentiation
18	IL-10 Signaling
19	Gαq Signaling
20	Ephrin Receptor Signaling
21	AMPK Signaling
22	IL-6 Signaling
23	Pathogen Induced Cytokine Storm Signaling Pathway
24	IL-17 A Signaling
25	IL-12 Signaling and Production in Macrophages
26	IL-17 Signaling
27	Glucocorticoid Receptor Signaling

**Table 8:** Signaling pathways associated with morphine and HIV in the PFC

Sl No:	Signaling Pathways-PFC
1	Neurovascular Coupling Signaling Pathway
2	Neuroinflammation Signaling Pathway
3	GABA Receptor Signaling
4	CREB Signaling in Neurons
5	S100 Family Signaling Pathway
6	Phagosome Formation
7	G-Protein Coupled Receptor Signaling
8	Gαi Signaling
9	cAMP-mediated signaling
10	nNOS Signaling in Neurons
11	Dopamine-DARPP32 Feedback in cAMP Signaling
12	Reelin Signaling in Neurons
13	Opioid Signaling Pathway
14	Glutamate Receptor Signaling
15	Serotonin Receptor Signaling
16	Neuropathic Pain Signaling In Dorsal Horn Neurons
17	Gαq Signaling
18	Synaptic Long-Term Potentiation
19	AMPK Signaling
20	Ephrin Receptor Signaling

### Comparison of signaling pathways in the Str and PFC of placebo treated HIV-1Tg rats revealed no gender-dependent changes in the enrichment of signaling of pathways

**Fig. 7:**
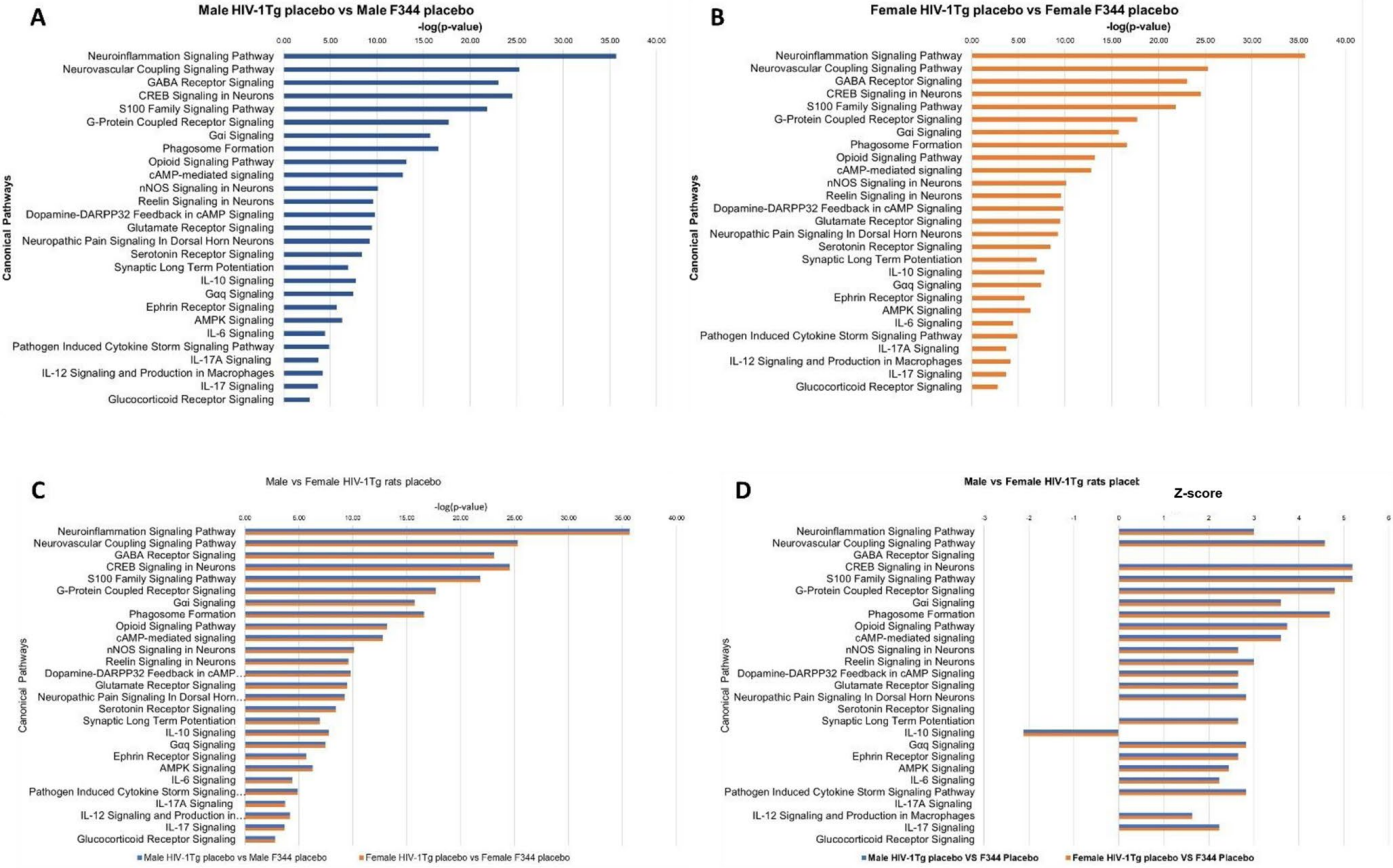
Signaling pathway enriched in response to HIV alone. (**A**) Male HIV-1Tg placebo, (**B**) Female HIV-1Tg placebo, (**C**) comparison of p-value of overlap Male vs Female HIV-1Tg placebo, (**D**) comparison of z-score between Male vs Female HIV-1Tg placebo

The influence of HIV alone on the signaling pathways are shown in Fig. 7. The p-value (-log(p-value)) of overlap of the 26 signaling pathways in the Str of both male and female HIV-1Tg rats received placebo pellets. p-value of overlap is the measure of likelihood that the association between set of genes in our dataset and a given pathway is due to random chance. The smaller the P-value indicates the corresponding pathway is significantly associated with the uploaded dataset. All the signaling pathways were found to be significantly enriched in both male (Fig. 7A) and female (Fig. 7B) HIV-1Tg rats received placebo pellets. We then compared the p-value of overlap of the signaling pathways in both male and female HIV-1Tg rats received placebo. The p-value of overlap for the signaling pathways were found to be same in the placebo treated HIV-1Tg rats, suggesting no gender-dependent changes in the enrichment of signaling pathways (Fig. 7C). IPA also predicted the activation state of the signaling pathways. All the signaling pathways excluding IL-10 signaling pathway were found to be activated, IL10 signaling pathway was found to be inhibited (Fig. 7D).

**Fig. 8:**
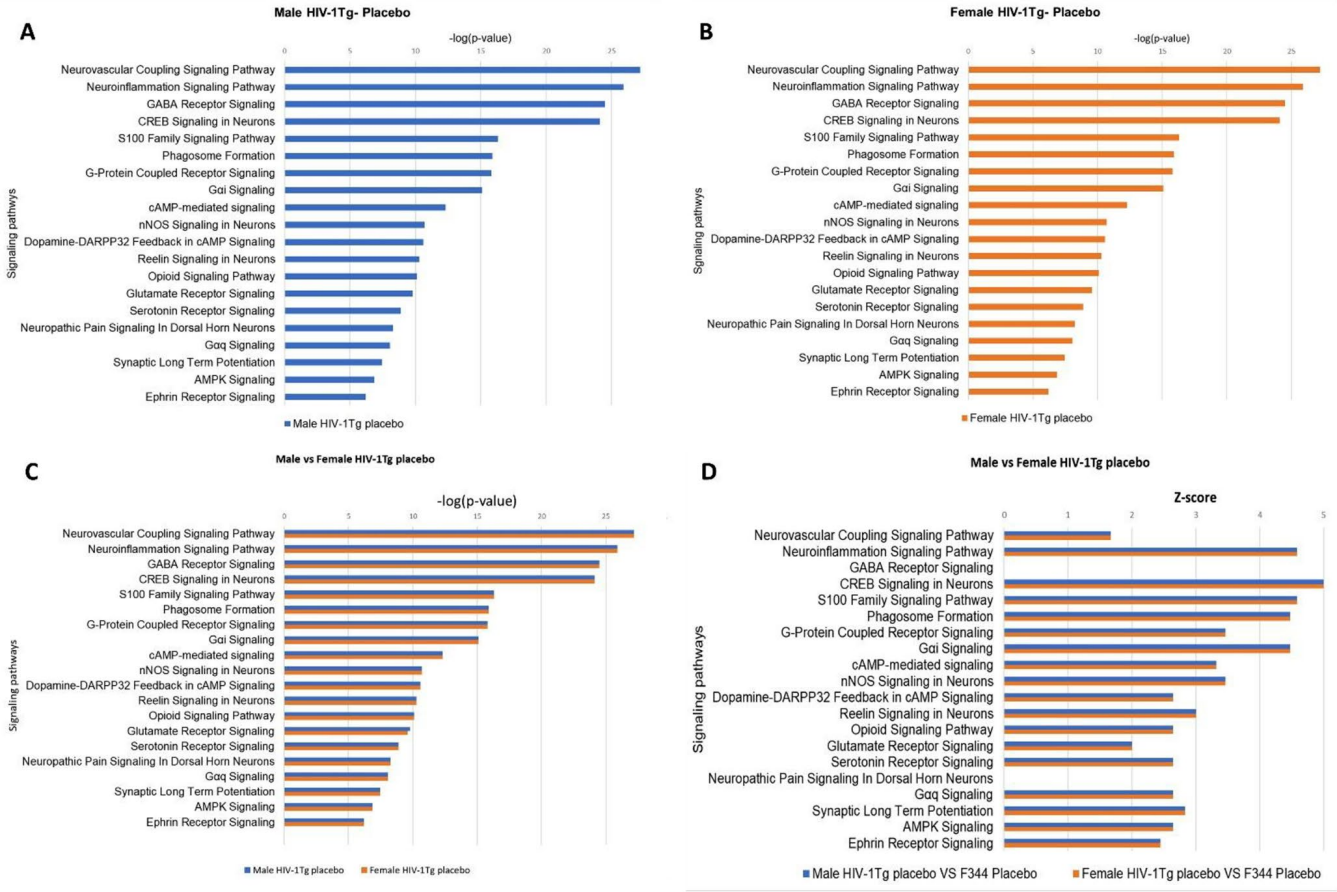
p-value of overlap and z-score of the 20-morphine addiction/dependance associated signaling pathways in the PFC in both Male and Female HIV-1Tg rats received placebo pellets. (**A**) Male HIV-1Tg placebo, (**B**) Female HIV-1Tg placebo; (**C**) Male vs Female HIV-1Tg placebo, (**D**) comparison of z-score between Male vs Female HIV-1Tg placebo

Figure 8 shows the p-value (-log(p-value) of overlap of the 20-morphine addiction/dependance associated signaling pathways in the PFC in both male and female HIV-1Tg rats received placebo pellets. All the 20 signaling pathways were found to be significantly enriched in both male (Fig. 8A) and female (Fig. 8B) HIV-1Tg rats received placebo pellets. We then compared the p-value of overlap of the signaling pathways in both male and female HIV-1Tg rats received placebo. Our comparison analysis revealed that there is no gender-dependent change in the p-value of overlap between placebo-treated male and female HIV-1Tg rats (Fig. 8C). The z-scores for all these 20 signaling pathways were found to be positive suggesting that these pathways are strongly activated in HIV-1Tg rats (Fig. 8D).

### Gender-dependent changes in the enrichment of signaling pathways in HIV-1Tg rats given morphine treatment

**Fig. 9:**
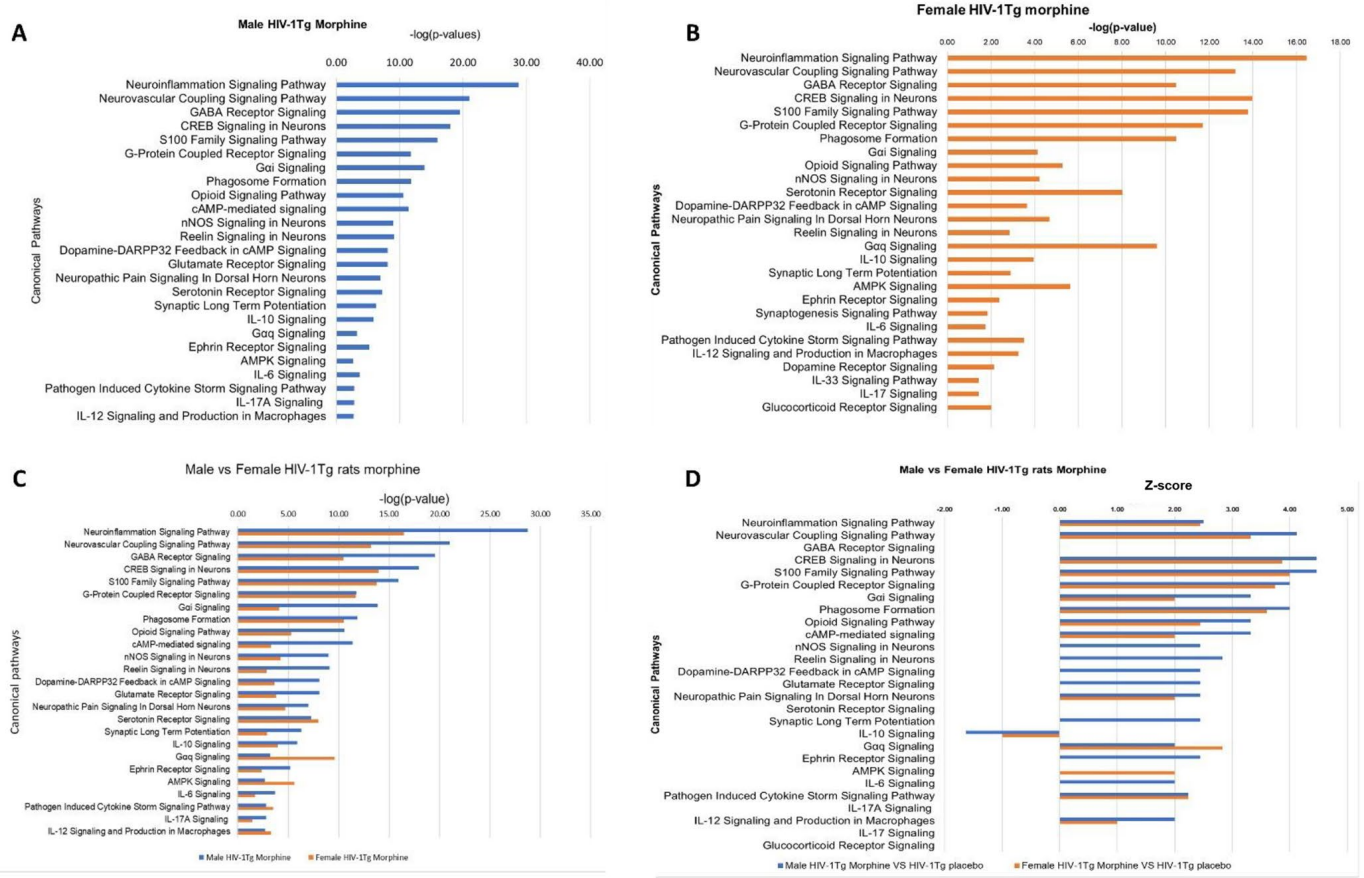
p-value of overlap and activation state of the signaling pathways in the striatum of HIV transgenic rats received morphine pellets: (**A**) Male HIV-1Tg rats (**B**) Female HIV-1Tg rats (**C**) gender-based comparison of the signaling pathways (**D**) activation state of signaling pathways, positive z-score indicates activation and negative indicates inhibition

The 26 signaling pathways associated with morphine addiction/dependance were also found to be significantly (*p* < 0.05) enriched in the striatum of morphine treated HIV-1Tg rats. Neuroinflammation signaling pathway was found to be the top enriched signaling pathway in both male (p-value 1.71E-29) and female (p-value 3.37E-17) HIV-1Tg rats (Fig. 9A and B). The p-value of overlap was then used to compare the degree of enrichment of signaling pathways in male and female HIV-1Tg rats. We found that, p-value for majority of the signaling pathways including Neuroinflammation Signaling Pathway, Neurovascular Coupling Signaling Pathway, GABA Receptor Signaling, CREB Signaling in Neurons, S100 Family Signaling Pathway, G-Protein Coupled Receptor Signaling, Gαi Signaling, Phagosome Formation, Opioid Signaling Pathway, cAMP Signaling, nNOS Signaling in Neurons, Reelin signaling, Dopamine-DARPP32 Feedback in cAMP Signaling, Glutamate Receptor Signaling, Neuropathic Pain Signaling In Dorsal Horn Neurons, Synaptic Long Term Potentiation, IL-10 Signaling, Ephrin Receptor Signaling, IL-6 Signaling, and IL-17 A Signaling was found to be lower male HIV-1Tg rat compared to females, suggesting high enrichment of these pathways in males. On the other side, p-value Serotonin receptor signaling, Gαq signaling, AMPK signaling, and Pathogen Induced Cytokine Storm Signaling Pathway was found to be lower in female rats compared to males (Fig. 9C). The activation state of the signaling pathways were determined using the z-score. Signaling pathways including Neuroinflammation Signaling Pathway, Neurovascular Coupling Signaling Pathway, GABA Receptor Signaling, CREB Signaling in Neurons, S100 Family Signaling Pathway, G-Protein Coupled Receptor Signaling, Gαi Signaling, Phagosome Formation, Opioid Signaling Pathway and cAMP-mediated signaling had a positive z-score, suggesting their activation in both morphine treated male and female HIV-1Tg rats. Whereas IL10 signaling was found to be inhibited in both male and female HIV-1Tg rats. Signaling pathways like nNOS Signaling in Neurons, Reelin Signaling in Neurons, Dopamine-DARPP32 Feedback in cAMP Signaling, Glutamate Receptor Signaling, Neuropathic Pain Signaling In Dorsal Horn Neurons, Serotonin Receptor Signaling, Synaptic Long Term Potentiation, IL-10 Signaling, Gαq Signaling, Ephrin Receptor Signaling, AMPK Signaling, and IL-6 Signaling were found to be activated only in male HIV-1Tg rats, in females, the activation state of these pathways were unpredicted. Similarly, AMPK signaling was fond to be activated only in female HIV-1Tg rats. The activation state of GABA Receptor Signaling, Serotonin Receptor Signaling IL-17 A Signaling, IL-12 Signaling and Production in Macrophages IL-17 Signaling and Glucocorticoid Receptor Signaling was unpredicted in both male and female HIV-1Tg rats (Fig. 9D).

**Fig. 10:**
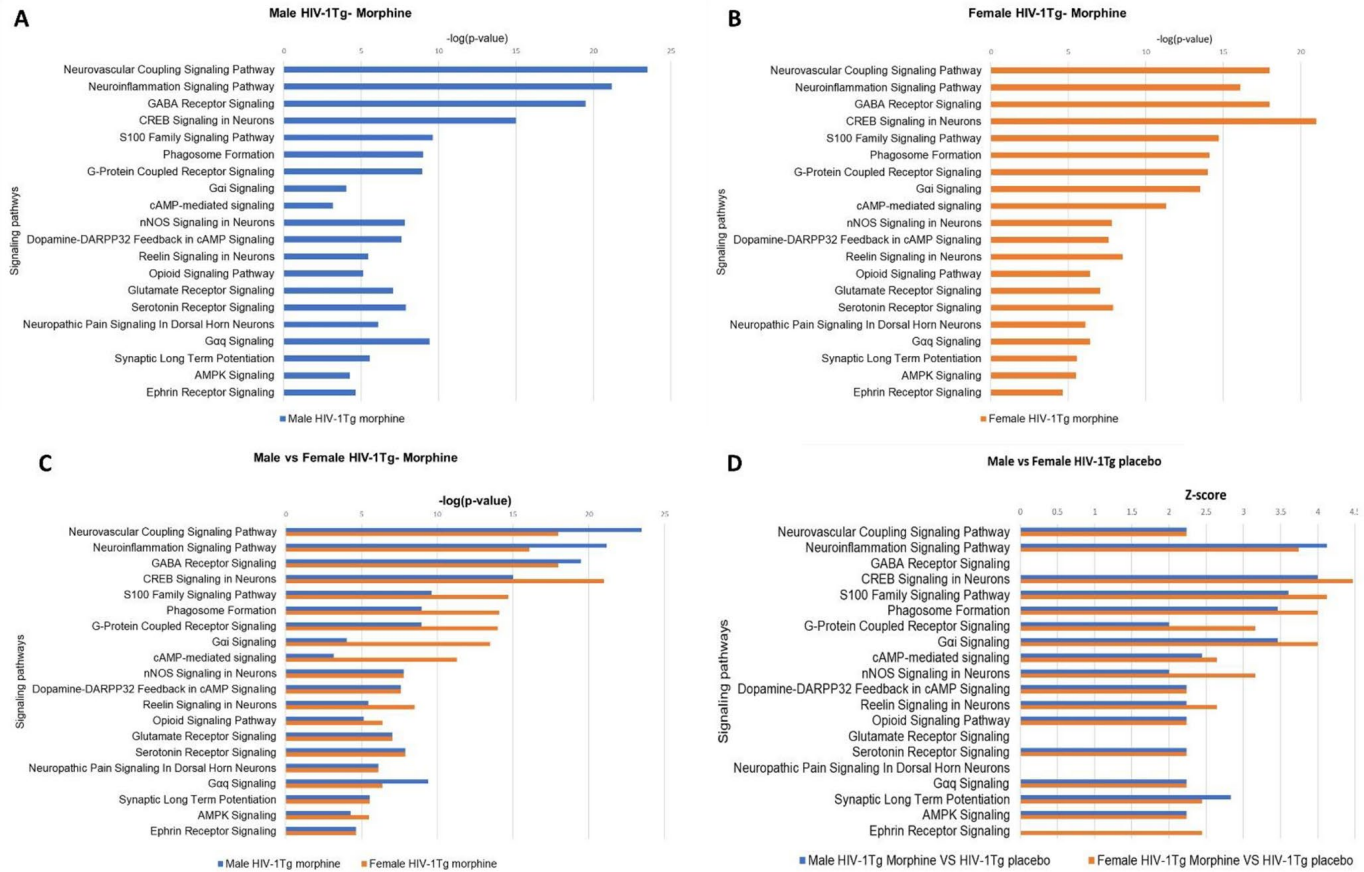
p-value of overlap and activation state of the signaling pathways in the PFC HIV transgenic rats received morphine pellets: (**A**) male HIV-1Tg rats (**B**) female HIV-1Tg rats (**C**) gender-based comparison of the signaling pathways (**D**) activation state of signaling pathways, positive z-score indicates activation and negative indicates inhibition

All the 20 signaling pathways associated with morphine addiction/dependance were also found to be significantly (*p* < 0.05) enriched in the PFC of both male (Fig. 10A) and female (Fig. 10B) morphine treated HIV-1Tg rats. The p-value of overlap was then used to compare the degree of enrichment of signaling pathways in male and female HIV-1Tg rats. We found that, among the 20 signaling pathways, 9 signaling pathways were highly enriched (indicated by strong p-value) in morphine treated female HIV-1Tg rats. These include CREB Signaling in Neurons, S100 Family Signaling Pathway, Phagosome Formation, G-Protein Coupled Receptor Signaling, Gαi Signaling, cAMP-mediated signaling, Reelin Signaling in Neurons, Opioid Signaling Pathway and AMPK Signaling. Four signaling pathways including Neurovascular Coupling Signaling Pathway, Neuroinflammation Signaling Pathway, GABA Receptor Signaling and Gαq Signaling were found to be highly enriched in male HIV-1Tg rats. However, both male and HIV-1Tg rats exhibited similar p-value of overlap for nNOS Signaling in Neurons, Dopamine-DARPP32 Feedback in cAMP Signaling, Glutamate Receptor Signaling, Serotonin Receptor Signaling, Neuropathic Pain Signaling in Dorsal Horn Neurons, Synaptic Long-Term Potentiation and Ephrin Receptor Signaling (Fig. 10C). Majority of the signaling pathways exhibited a positive z-score, suggesting their activation in both the male and female HIV-1Tg rats. However, z-scores for GABA Receptor Signaling, Glutamate Receptor Signaling pathway, Neuropathic Pain Signaling in Dorsal Horn Neurons in both male and female HIV-1Tg rats, and Ephrin receptor signaling in male HIV-1Tg rats were undetermined (Fig. 10D).

### Comparison of signaling pathways in morphine-treated F344 vs. HIV-1Tg rats

**Fig. 11:**
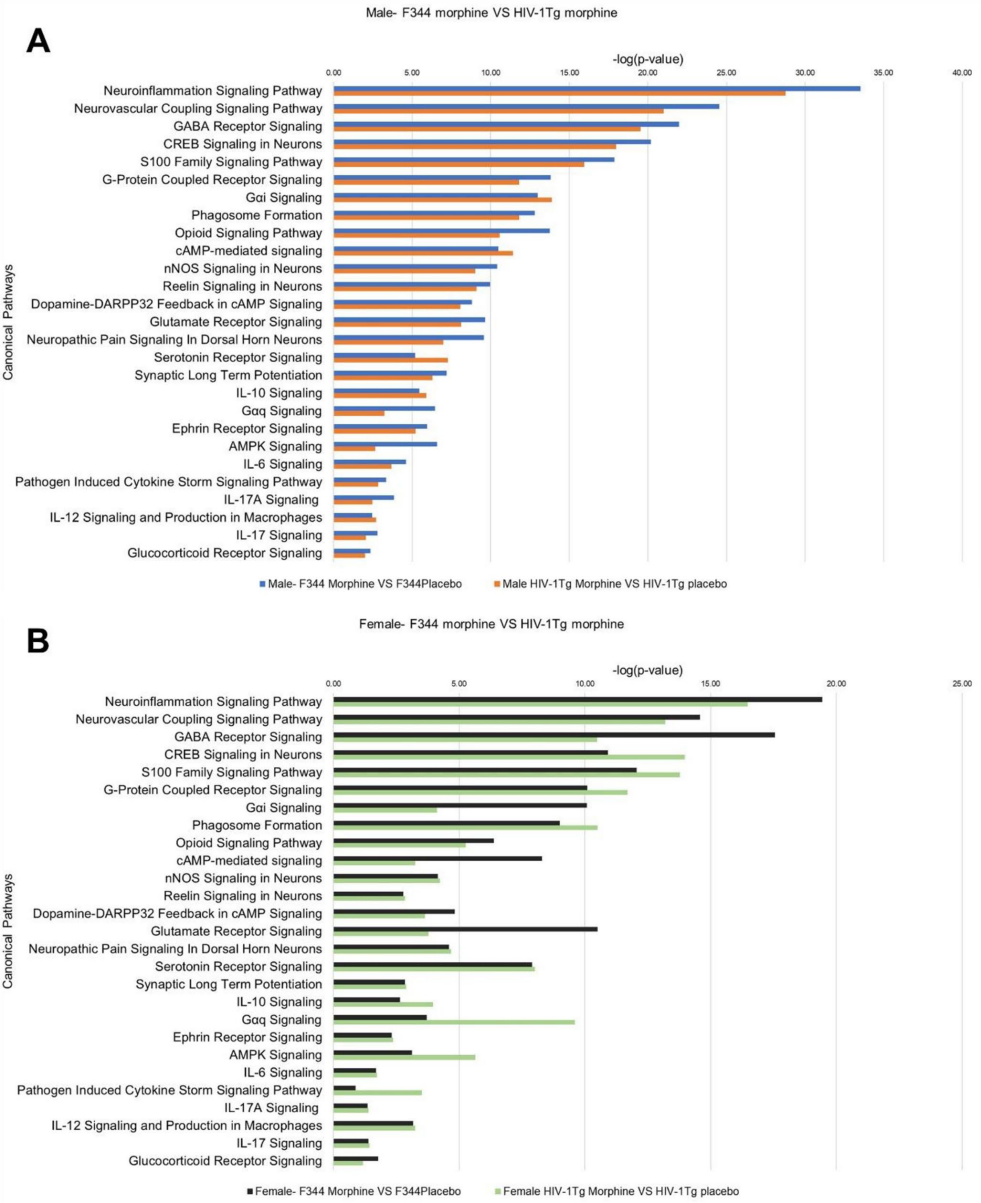
Combined effect of morphine and HIV on the enrichment of signaling pathways in the striatum. (**A**) Male F344 vs Male HIV-1Tg rats (**B**) Female F344 vs Female HIV-1Tg rats

**Fig. 12:**
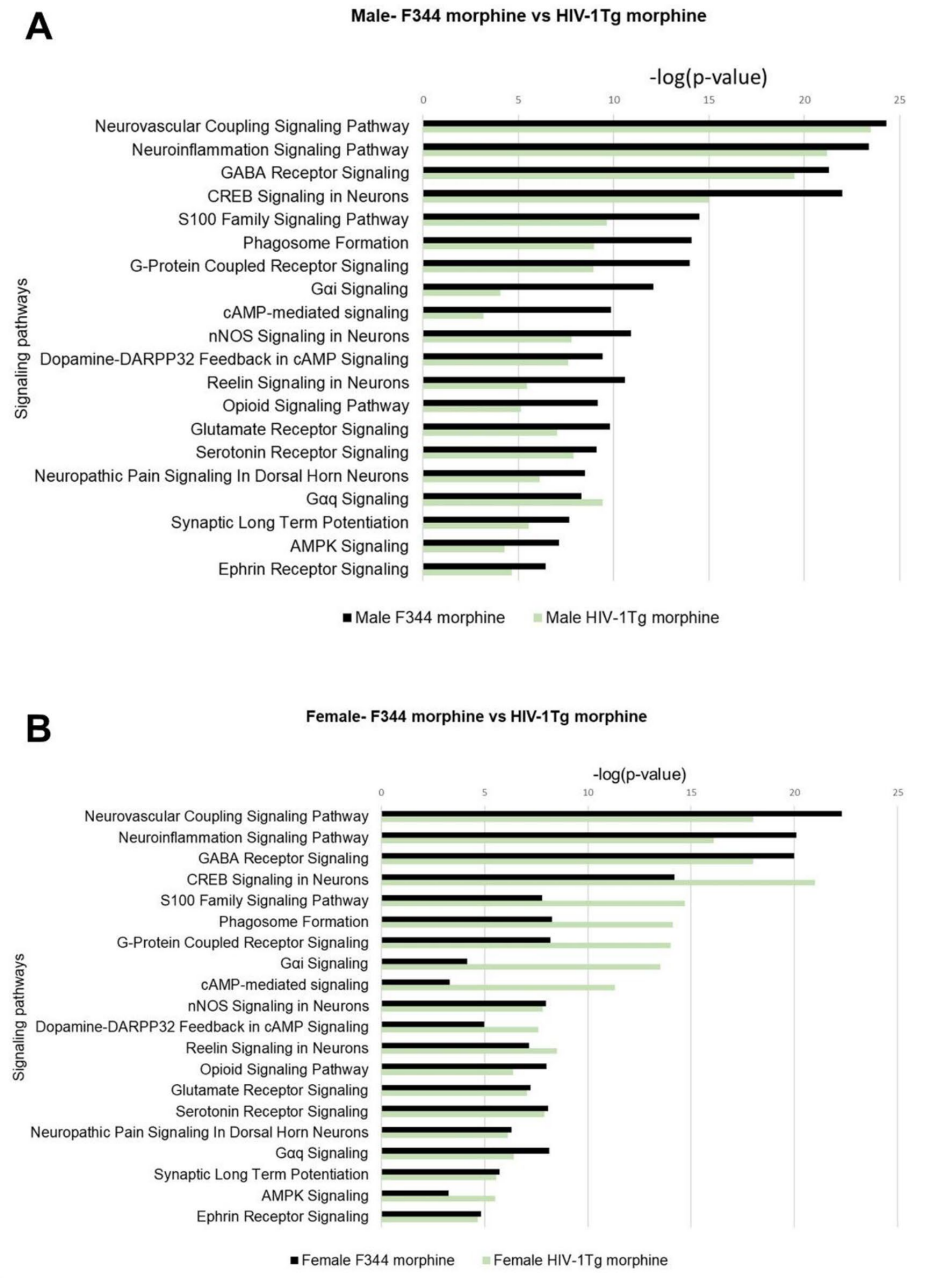
Combined effect of morphine and HIV on the enrichment of signaling pathways in the PFC. (**A**) Male F344 vs Male HIV-1Tg rats (**B**) Female F344 vs Female HIV-1Tg rats

To study the combined effect of morphine and HIV on the signaling pathways, we compared the p-vale of overlap between F344 rats and HIV-1Tg rats received morphine pellets (Fig. 11). In Str of males rats, the majority of the signaling pathways were highly significant in F344 rats received morphine pellets as indicated by low p-values. However, some of the key signaling pathways associated with morphine signaling, including Gαi Signaling, cAMP-mediated signaling, serotonin signaling and IL10 signaling, were found to be highly enriched in HIV-1Tg rats compared to F344 rats (Fig. 11A). In Str of female rats among the 27 signaling pathways, 18 pathways were found to be highly enriched in morphine treated F344 rats, 9 signaling pathway were found to be enriched in HIV-1Tg rats received morphine pellets (Fig. 11B). The combined effects of HIV and morphine were also studied in the PFC by comparing the p-value of overlap of the signaling pathways between morphine treated F344 and HIV-1Tg rats (Fig. 12). In males, among the 20 signaling pathways, 19 pathways including Neurovascular Coupling Signaling Pathway, Neuroinflammation Signaling Pathway, GABA Receptor Signaling, CREB Signaling in Neurons, S100 Family Signaling Pathway, Phagosome Formation, G-Protein Coupled Receptor Signaling, Gαi Signaling, cAMP-mediated signaling, nNOS Signaling in Neurons, Dopamine-DARPP32 Feedback in cAMP Signaling, Reelin Signaling in Neurons, Opioid Signaling Pathway, Glutamate Receptor Signaling, Serotonin Receptor Signaling, Neuropathic Pain Signaling In Dorsal Horn Neurons, Synaptic Long Term Potentiation, AMPK Signaling and Ephrin Receptor Signaling were highly enriched in F344 rats. The p-value of overlap for Gαq signaling was stronger in HIV-1Tg rats compared to F344 rats (Fig. 12A). In females, 11 signaling pathways, Neurovascular Coupling Signaling Pathway, Neuroinflammation Signaling Pathway, GABA Receptor Signaling, Opioid Signaling Pathway, Glutamate Receptor Signaling, Serotonin Receptor Signaling, Neuropathic Pain Signaling In Dorsal Horn Neurons, Gαq signaling, Synaptic Long Term Potentiation, and Ephrin receptor signaling were highly enriched in F344 rats, whereas 9 signaling pathways including CREB signaling neurons, S100 family signaling pathway, phagosome formation, G-Protein Coupled Receptor Signaling, Gαi Signaling, cAMP-mediated signaling, Dopamine-DARPP32 Feedback in cAMP Signaling, Reelin signaling in neurons, and AMPK signaling were found to be highly enriched in HIV-1Tg rats (Fig. 12B).

## Discussion

In the present study, we aimed to study the interplay of signaling pathways leading to increased opioid dependance and addiction in HIV-1Tg rats. We have previously reported that MOR expression level is significantly higher in HIV-1Tg rats than the control rats (Chang et al. 2007). Misuse of opioid substances, particularly morphine, is a major comorbidity in human immunodeficiency virus (HIV) patients. HIV patients are known to use more opioids and more likely to have opioid use disorders (Hodder et al. 2021; Vigorito et al. 2015). Also, HIV patients are more prone to chronic pain, and they receive higher doses of morphine compared to the general population, putting them at high risk of developing opioid related disorders. It is suggested that the enhanced neurological abnormalities and opioid addiction seen in HIV patients involve the interplay among signaling pathways (Chang 2013; Kosten and George 2002).

Morphine administration stimulates MOR localization at the Ventral Tegmental Area (VTA) and stimulates dopaminergic neurons to release dopamine to ventral Str and prefrontal cortex (PFC), leading to neuroadaptations (Listos et al. 2019). In the absence of morphine, it leads to tolerance, addiction and dependance (Kosten and George 2002; Listos et al. 2019). However, the impact of HIV viral proteins on morphine addiction and dependance is less explored. Here we treated both male and female F344 and HIV-1 transgenic (HIV-1Tg) rats with morphine pellets and sacrificed them at morphine tolerance to study the gene expression changes among the opioid dependance genes and to identify the signaling pathways associated with them. HIV-1Tg rats have been a well-accepted rodent model mimicking the HIV patients under combination Antiretroviral Therapy (cART) (Vigorito et al. 2015). The HIV-1Tg model was developed as a non-infectious model that was developed by deleting the gag and pol genes, thereby inhibiting viral assembly and replication (Reid et al. 2001).

Our data showed that, in response to morphine, in Str, among the 65 opioid dependance genes, 12 genes in male F344 rats (Table 3A) and 15 genes in male HIV-1Tg rats (Table 4A) were significantly upregulated. Whereas, in females, only 4 genes in F344 rats (Table 3B) and one gene in HIV-1Tg rats (Table 4B) were significantly upregulated. In PFC, 17 genes in male F344 rats (Table 5A) and 11 genes in male HIV-1Tg rats (Table 6A) were significantly upregulated. In females, 7 genes in F344 rats (Table 5B) and 8 genes in HIV-1Tg rats (Table 6B) were significantly upregulated. Considering that the sample size for this study was *n* = 3, we decided not to exclude the genes with p-value < 0.05. We employed the IPA core analysis to identify the enriched signaling pathways, upstream regulators and functions associated with 65 opioid dependance genes based on their fold change values and p-values (Krämer et al. 2014; Yu et al. 2016). We identified 26 signaling pathways associated with morphine addiction/dependence in the Str and 20 signaling pathways in the PFC.

Summary of our core analysis emphasized two signaling pathways- Neuroinflammation signaling pathway and CREB signaling in neurons that were found to be activated in all morphine and HIV conditions (Figs. 3 and 4). HIV infected glial cells release viral proteins, inflammatory cytokines and chemokines leading to neuroinflammation (Garvey et al. 2014; Sil et al. 2020). Substance abuse, including morphine, has also been associated with neuroinflammation. Morphine itself triggers neuroinflammation by acting on TLR4 pathways in glial cells (Malik and Agrewala 2024), morphine’s interaction with HIV viral proteins were found to activate inflammasomes and trigger neuroinflammation (Shi et al. 2019). The heightened neuroinflammation in response to HIV viral proteins and morphine may impair reward circuits resulting in heightened dependance and addiction in PWH. Our findings also suggest similar mechanisms, activation of neuroinflammation signaling pathway in response to HIV viral proteins and morphine suggests chronic inflammatory reactions in both microglia and astrocytes of morphine treated HIV-1Tg rats. In microglia, IPA’s suggests morphine treatment results increased ROS production, oxidative stress, amyloid beta production, astrogliosis and neuronal damage. Inflammatory reactions in astrocytes resulted in increased T cell recruitment, Ca^2+^ signaling and neuronal apoptosis (Supplementary, S1). Ligand binding to the G-protein coupled receptor activates CREB phosphorylation and the activation of CREB signaling pathway in Neurons. CREB is involved in the formation of neuronal plasticity in the mesolimbic reward system including VTA, Str and PFC which results in neuroadaptations and results in addiction and dependance (Langlois and Nugent 2017; Nestler 2001). CREB signaling also regulates the gene expression of molecules like dynorphin in various brain regions including VTA, Str and PFC which mediates the negative effects of morphine (Barrot et al. 2002; Muschamp and Carlezon 2013; Nestler 2001).

In the Str, the enrichment of these signaling pathways in both male and female HIV-1Tg rats received placebo exhibited similar p-values, suggesting that no gender-dependent changes in the enrichment and the magnitude of activation (z-score) in HIV-1Tg rats (Fig. 7C and D). In the Str of morphine treated HIV-1Tg rats, we found gender-dependent changes in the enrichment and the activation state of the signaling pathways (Fig. 9C and D). Lower p-values of overlap [presented as higher -log (p-value) in Figs. 7, 9 and 11] for enrichment and higher z-score for the activation were observed in male HIV-1Tg rats compared to females. The nucleus of accumbens in the ventral Str is more prone towards the determinantal effects of HIV viral proteins (Paul et al. 2005). Studies have reported that HIV viral proteins induce neurochemical alterations and dopaminergic signaling in the Str (Bertrand et al. 2018). Chronic exposure resulted in loss of dopaminergic nerve terminals (Gelman et al. 2012), loss of dendritic spine integrity and plasticity (Roscoe et al. 2014).

PFC is also an integral part of the mesolimbic reward system; it provides feedback to the VTA that helps to overcome the drives to pleasure through unsafe actions. This feedback is disrupted during addiction (Kosten and George 2002). Our pathway comparison analysis revealed that the majority of the signaling pathways were enriched in female HIV-1Tg rats (Figs. 6, 8 and 10). This includes some key pathways like CREB Signaling in Neurons, Gαi Signaling, cAMP-mediated signaling, Reelin Signaling in Neurons, Opioid Signaling Pathway and AMPK Signaling. Dysregulated neuronal signaling in the PFC is associated with HIV infection (Ferris et al. 2008). Tat viral protein increases Ca^2+^ influx in the pyramidal neurons on the PFC leading to neurotoxicity (Brailoiu et al. 2008). Neurotoxic effects of Tat in the PFC can be enhanced by other addictive substance like cocaine (Aksenov et al. 2006). Another study have reported that Tat mediated hyper excitability of PFC neurons is enhanced during cocaine self-administration (Wayman et al. 2016). Impairment in the neuronal signaling in the PFC and impairment of the frontostriatal connectivity results in compulsive drug intake cognitive deficits in neuroHIV (Ipser et al. 2015; Plessis et al. 2014; Volkow and Morales 2015). Ipser et al. have reported that HIV attenuates the connectivity between dorsolateral prefrontal cortex (DLPFC) and dorsal caudate and they also report impaired connectivity between dorsal caudate and central executive network in male HIV patients compared to control participants (Ipser et al. 2015). To the best of our knowledge, we did not find any other study that focused on the signaling pathways associated with morphine and HIV. Our study is the first to investigate the signaling pathways in the Str and PFC associated with morphine and HIV, which could contribute to enhanced addiction, dependence, and other neurological abnormalities.

We also found that certain pathways in theStr including Gαi Signaling, cAMP-mediated signaling, serotonin signaling and IL10 signaling were found to be highly enriched in HIV-1Tg rats compared to F344 rats. It has been well established that MOR couples with Gαi/o subunit for its inhibitory effects on CAMP formation (Lamberts and Traynor 2013). It has been previously reported that HIV binding activates Gαi Signaling in CXCR4 receptors thus in turn inhibiting adenylyl cyclase and reducing CAMP levels (Ganju et al. 1998; Wu and Yoder 2009). Signaling in the chemokine receptors are also well studied in morphine’s action. It has been reported that treatment with chemokine receptor antagonists significantly shifts morphine’s dose response to the left (Eisenstein et al. 2020). Targeting chemokine receptors can be a novel therapeutic approach to reduce morphine’s dose and negative effects. In line with these reports, our findings suggested that morphine in HIV patients could induce altered Gαi Signaling and cAMP-mediated signaling, which in turn leads to increased opioid addiction and dependance in HIV patients. Our study also revealed a strong inhibition of anti-inflammatory IL-10 signaling pathway in HIV-1Tg rats. IL-10 inhibits MHC class II formation and CD80-86 interaction in both macrophages and monocytes thereby inhibiting cytokine release (Rousset et al. 1992). Previous studies have reported increased IL-10 mRNA expression in HIV patients. Inhibition of IL-10 signaling in HIV patients results in increased HIV-specific CD4 cell, CD8 T-cell proliferation and cytokine secretion (Brockman et al. 2009). Chronic morphine treatment has been found to inhibit IL-10 signaling and activate IL-12 signaling in human dendritic cells (DC) thereby promoting DC maturation and inflammation (Messmer et al. 2006). Taken together, morphine inhibitory effects on IL-10 signaling can aggravate inflammatory reaction and this can be one of the potential mechanisms of inflammatory reactions in HIV-1Tg rats at morphine tolerance.

Taken together, our study revealed the overlapping signaling pathways in the Str and PFC that were involved in morphine’s addiction and dependance. Our IPA analysis identified Neuroinflammation signaling pathway and CREB signaling in neurons as the top signaling pathway activated in morphine-treated F344 and HIV-1Tg rats. We found gender-dependent enrichment of signaling pathways in the Str and PFC. In Str, signaling pathways were highly enriched in the HIV-1Tg rats, whereas pathway enrichment in PFC was higher in female HIV-1Tg rats. In Str, signaling pathways like Gαi Signaling, cAMP-mediated signaling, serotonin signaling and IL10 signaling were found to be highly enriched in male HIV-1Tg rats compared to control F344 rats.

## Electronic supplementary material

Below is the link to the electronic supplementary material.

Supplementary Material 1

## Data Availability

No datasets were generated or analysed during the current study.
